# Estimating a Preference-Based Value Set for the Mental Health Quality of Life Questionnaire (MHQoL)

**DOI:** 10.1177/0272989X231208645

**Published:** 2023-11-19

**Authors:** Frédérique C. W. van Krugten, Marcel F. Jonker, Sebastian F. W. Himmler, Leona Hakkaart-van Roijen, Werner B. F. Brouwer

**Affiliations:** Erasmus School of Health Policy & Management, Erasmus University Rotterdam (ESHPM), Rotterdam, The Netherlands; Erasmus Centre for Health Economics Rotterdam (EsCHER), Erasmus University Rotterdam, Rotterdam, The Netherlands; Erasmus Choice Modelling Centre (ECMC), Erasmus University Rotterdam, Rotterdam, The Netherlands; Erasmus School of Health Policy & Management, Erasmus University Rotterdam (ESHPM), Rotterdam, The Netherlands; Erasmus Centre for Health Economics Rotterdam (EsCHER), Erasmus University Rotterdam, Rotterdam, The Netherlands; Erasmus Choice Modelling Centre (ECMC), Erasmus University Rotterdam, Rotterdam, The Netherlands; Erasmus School of Health Policy & Management, Erasmus University Rotterdam (ESHPM), Rotterdam, The Netherlands; Erasmus Centre for Health Economics Rotterdam (EsCHER), Erasmus University Rotterdam, Rotterdam, The Netherlands; Erasmus Choice Modelling Centre (ECMC), Erasmus University Rotterdam, Rotterdam, The Netherlands; Erasmus School of Health Policy & Management, Erasmus University Rotterdam (ESHPM), Rotterdam, The Netherlands; Erasmus Centre for Health Economics Rotterdam (EsCHER), Erasmus University Rotterdam, Rotterdam, The Netherlands; Erasmus School of Health Policy & Management, Erasmus University Rotterdam (ESHPM), Rotterdam, The Netherlands; Erasmus Centre for Health Economics Rotterdam (EsCHER), Erasmus University Rotterdam, Rotterdam, The Netherlands

**Keywords:** utility measurement, discrete choice experiment, quality of life, mental health, MHQoL

## Abstract

**Background:**

Health economic evaluations using common health-related quality of life measures may fall short in adequately measuring and valuing the benefits of mental health care interventions. The Mental Health Quality of Life questionnaire (MHQoL) is a standardized, self-administered mental health–related quality of life instrument covering 7 dimensions known to be relevant across and valued highly by people with mental health problems. The aim of this study was to derive a Dutch value set for the MHQoL to facilitate its use in cost-utility analyses.

**Methods:**

The value set was estimated using a discrete choice experiment (DCE) with duration that accommodated nonlinear time preferences. The DCE was embedded in a web-based self-complete survey and administered to a representative sample (*N* = 1,308) of the Dutch adult population. The matched pairwise choice tasks were created using a Bayesian heterogeneous D-efficient design. The overall DCE design comprised 10 different subdesigns, with each subdesign containing 15 matched pairwise choice tasks. Each participant was asked to complete 1 of the subdesigns to which they were randomly assigned.

**Results:**

The obtained coefficients indicated that “physical health,”“mood,” and “relationships” were the most important dimensions. All coefficients were in the expected direction and reflected the monotonic structure of the MHQoL, except for level 2 of the dimension “future.” The predicted values for the MHQoL ranged from −0.741 for the worst state to 1 for the best state.

**Conclusions:**

This study derived a Dutch value set for the recently introduced MHQoL. This value set allows for the generation of an index value for all MHQoL states on a QALY scale and may hence be used in Dutch cost-utility analyses of mental healthcare interventions.

**Highlights:**

- A discrete choice experiment was used to derive a Dutch value set for the MHQoL.
- This allows the use of the MHQoL in Dutch cost-utility analyses.
- The dimensions physical health, mood, and relationships were the most important.
- The utility values range from −0.741 for the worst state to 1 for the best state.

Mental health problems, such as depression and anxiety, affect 1 in 4 people at some point in their lives and can have a large impact on people’s health, functioning, and quality of life.^1^ Apart from the substantial impact on an individual level, as well as on the family level,^2^ the societal burden of mental health problems is profound. Mental health problems are, for instance, associated with substantial health care costs, productivity losses, and elevated costs related to the educational and criminal justice systems.^3–5^ Given the large burden of mental health problems, both on an individual and on a societal level, national health systems strive to deliver accessible and high-quality mental health care. Given the limited available health care resources, it is, however, essential that resources are used in an efficient way, so as to produce as much (health) benefits as possible.

Health economic evaluations are used to identify the most efficient way to use available resources and are increasingly employed to inform decisions on the collective reimbursement of health care interventions.^6^ A frequently used and often recommended type of economic evaluation is cost-utility analysis,^7^ in which health benefits are expressed as quality-adjusted life-years (QALYs). The QALY combines length of life (i.e., the number of remaining years a person is expected to live) and health-related quality of life experienced during those years. The most commonly used method for measuring health-related quality of life and therefore to inform the “Q” in the QALY is through the administration of a generic preference-based instrument, such as the EuroQol 5-dimensional (EQ-5D) questionnaire^8^ and the 36-item Short-Form Health Survey (SF-36).^9^ It has, however, been questioned whether these generic quality of life instruments are sufficiently sensitive to effects of mental health problems. Therefore, the Mental Health Quality of Life questionnaire (MHQoL) was developed, which is a quality of life measure specifically designed for use in adults with mental health problems.^10^ The descriptive system of the MHQoL comprises 7 dimensions known to be relevant across and valued highly by people with mental health problems. This descriptive part of the MHQoL system was based on previous work carried out by Connell et al.^11,12^ that provided a comprehensive overview of the quality of life dimensions most relevant to people with mental health problems. After its development, the MHQoL was evaluated in terms of its psychometric properties in a heterogeneous population of mental health care service users and members from the general population. The MHQoL was demonstrated to have favorable psychometric properties and showed promise as a simple and effective tool for assessing quality of life in people with mental health problems.^10^ However, to date, a value set with standard scores to generate health state utility values for QALY calculations, making the MHQoL suitable for use in cost-utility evaluations, is lacking. The objective of this study, therefore, was to derive a Dutch value set for the MHQoL. The Dutch value set for the MHQoL was generated using a state-of-the-art discrete choice experiment (DCE) with duration approach that accommodated nonlinear time preferences. The generated value set represents the preferences of the Dutch general population and can be used to attach utility values to all 16,384 (4^7^) unique MHQoL states and may therefore be used in Dutch cost-utility analyses of mental health care interventions.

## Methods

### Elicitation Mode

Various preference-elicitation methods are available to derive the required preference weights necessary for cost-effectiveness calculations. Of the available preference-elicitation methods, ordinal valuation tasks such as DCEs are increasingly used for health state valuation.^13^ However, an important challenge when using DCEs as a health state valuation method is that traditional DCEs generate only relative values on a latent scale, ranging from the worst included health state to the best included health state. To be of use for cost-utility evaluations of health care interventions, it is, however, useful that health-state utility values reflect the relative desirability of unique health states on a QALY scale that is anchored at 0 (death) and 1 (full health). An increasingly used method to anchor health-state utility values on the QALY scale is through the inclusion of duration as an attribute in DCE choice tasks.^14,15^ The Dutch value set for the MHQoL was estimated using an efficient DCE with duration design that accommodated nonlinear time preferences.^16,17^ This DCE design has been demonstrated to keep the complexity of the duration tradeoffs manageable for participants^16,17^ and reduces the need for the adoption of simplifying choice heuristics (i.e., simplified decision-making strategies) by participants.^18,19^

### MHQoL Descriptive System

The MHQoL is a standardized, self-administered mental health–related quality of life instrument.^10^ The MHQoL consists of a descriptive system, the MHQoL-7D, and a visual analog scale, the MHQoL-VAS, which records the self-reported, subjective general psychological well-being on a horizontal scale ranging from zero (*worst imaginable psychological well-being*) to 10 (*best imaginable psychological well-being*). The descriptive system (see Table 1) was based on previous work carried out by Connell et al.^11,12^ and comprises 7 items covering the following 7 dimensions: self-image, independence, mood, relationships, daily activities, physical health, and future. Each item has 4 levels (e.g., ranging from *very satisfied* to *very dissatisfied*), and, hence the descriptive system can describe 16,384 (4^7^) unique states. The MHQoL descriptive system is shown in Table 1. The full MHQoL in Dutch, including the MHQoL-VAS, as administered to participants can be found in Appendix A.

**Table 1:** MHQoL Descriptive System (MHQoL-7D)^a^

Self-image	
SI1	I think very positively about myself
SI2	I think positively about myself
SI3	I think negatively about myself
SI4	I think very negatively about myself
Independence (e.g., freedom of choice, financial, co-decision making)	
IN1	I am very satisfied with my level of independence
IN2	I am satisfied with my level of independence
IN3	I am dissatisfied with my level of independence
IN4	I am very dissatisfied with my level of independence
Mood	
MO1	I do not feel anxious, gloomy, or depressed
MO2	I feel a little anxious, gloomy, or depressed
MO3	I feel anxious, gloomy, or depressed
MO4	I feel very anxious, gloomy, or depressed
Relationships (e.g., partner, children, family, friends)	
RE1	I am very satisfied with my relationships
RE2	I am satisfied with my relationships
RE3	I am dissatisfied with my relationships
RE4	I am very dissatisfied with my relationships
Daily activities (e.g., work, study, household, leisure activities)	
DA1	I am very satisfied with my daily activities
DA2	I am satisfied with my daily activities
DA3	I am dissatisfied with my daily activities
DA4	I am very dissatisfied with my daily activities
Physical health	
PH1	I have no physical health problems
PH2	I have some physical health problems
PH3	I have many physical health problems
PH4	I have a great many physical health problems
Future	
FU1	I am very optimistic about my future
FU2	I am optimistic about my future
FU3	I am gloomy about my future
FU4	I am very gloomy about my future

DA, daily activities; FU, future; IN, independence; MHQoL, Mental Health Quality of Life questionnaire; MO, mood; PH, physical health; RE, relationships; SI, self-image.

a The full MHQoL in Dutch, including the MHQoL-VAS, as administered to participants can be found in Appendix A.

### Study Sample

A representative online sample (*N* = 1,308) of the Dutch adult (18 y or older) population in terms of the distribution of age and sex in The Netherlands was recruited by Dynata, a commercial survey sample provider, using stratified sampling. The sample was larger than DCE valuation studies for other instruments^14^ and confirmed to be sufficient for the MHQoL using sample size calculations as described in the article by de Bekker-Grob et al.^20^ Participants were rewarded with points that could be redeemed against gift vouchers or donations to charities. The study was reviewed and approved by the Research Ethics Review Committee of the Erasmus School of Health Policy & Management, The Netherlands (reference number: 20-24), and digital informed consent was obtained from all participants.

### Discrete Choice Experiment

A DCE is a method designed to elicit stated preferences by asking participants to choose a preferred scenario from a selection of at least 2 scenarios. These scenarios are made up of characteristics, called attributes, which are varied by a prespecified range of categories, called levels, in a way that facilitates the derivation of the relative importance of the attributes and levels.^21^ Attributes and levels of the present DCE were defined by the descriptive system of the MHQoL (see Table 1). In addition to the 7 MHQoL attributes, a duration of life attribute with 17 levels (0.25, 0.5, 1, 2, 3, …, 14, 15 y) was added to facilitate tradeoffs between quantity and quality of life and hence to be able to place DCE estimates onto a QALY scale anchored on the states of death and full health. As none of the combinations of attribute levels were per definition considered conflicting, and to avoid an effect on the statistical efficiency of the DCE design, no constraints were applied on the occurrence of certain combinations of attribute levels.

**Figure 1:**
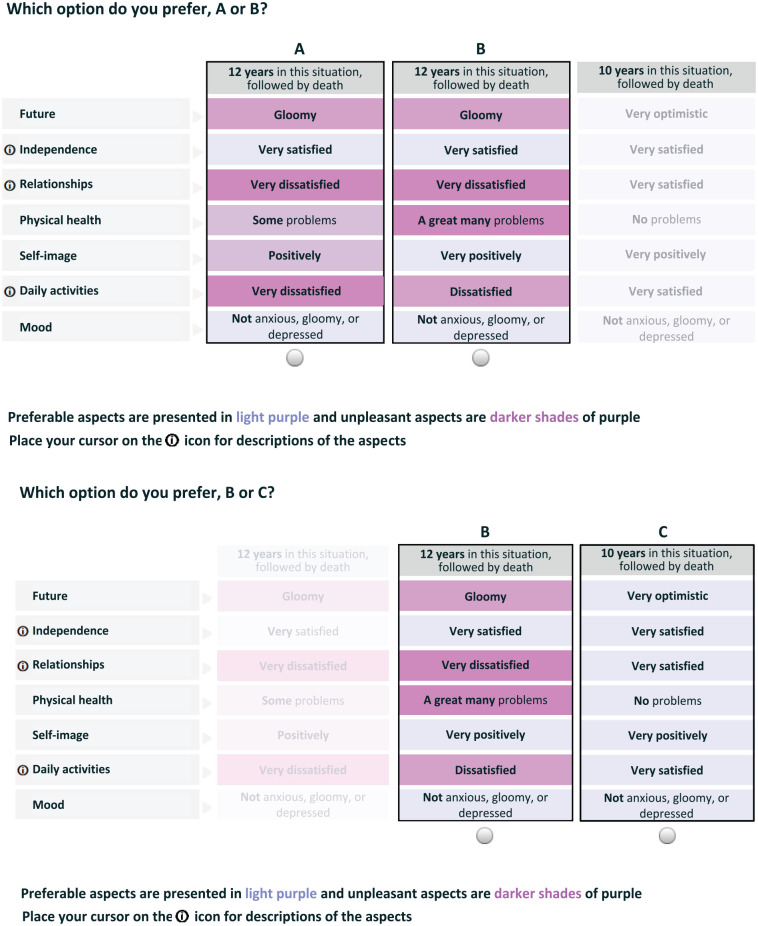
Visual representation of a matched pairwise choice task. (A) First of two matched pairwise choice tasks. (B) Second of two matched pairwise choice tasks.

To reduce the complexity of the choice tasks and hence to reduce the cognitive burden, dropout rates, and the adoption of simplifying choice heuristics by participants (e.g., attribute nonattendance), several measures were taken. First, the level descriptions were shortened and the attribute descriptions (when present) incorporated as a mouseover macro to an information icon. Second, matched pairwise choice tasks as introduced by Jonker et al.^17^ were used, in which, compared with more “traditional” DCE valuation formats, a simultaneous comparison between different impaired states and varying lengths of life was replaced by 2 choice tasks. In the first choice task, participants had to indicate which of 2 presented impaired states (A or B) with an equal duration of life they preferred. In the second, “matched” choice task, participants were asked to indicate if they preferred the impaired state (B) or a state in perfect health (i.e., without any problems on the MHQoL) but with a shorter duration of life (C). In addition, level overlap and color coding were applied to reduce the DCE task complexity.^18,19^ Level overlap constraints the number of attributes to be presented at the same level and was applied in each of the first of the 2 pairwise choice tasks. In these tasks, level overlap was imposed on 4 of the 7 MHQoL attributes; that is, 4 attributes were fixed at the same level and hence only 3 of 7 MHQoL attributes differed. Intensity color coding was applied to aid participants in signaling differences in attribute levels between the presented states.^17^ Level descriptions were color coded with shades of purple, with darker shades denoting worse MHQoL levels. See Figure 1 for a visual representation of a matched pairwise choice task, worded in English, as presented to participants.

### Experimental Design

Given the large descriptive system of the MHQoL and the imposed level overlap constraints, a so-called efficient DCE design was used to ensure adequate statistical efficiency. More specifically, a Bayesian heterogeneous D-efficient DCE design with 10 subdesigns and 15 pairwise choice tasks per subdesign was optimized using the TPC-QALY software package.^22^ This implied a simultaneous optimization of the efficiency of the 10 separate designs as well as the efficiency of the overall design. The D-efficiency criterion was calculated with 100 Bayesian draws, assuming an exponential discount function and based on the weighted average of the overall (i.e., combined) D-error (25% weight) and D-errors of the 10 subdesigns (75% weight). For the initial DCE design, default priors were used that reflected the monotonically decreasing preference for additional health problems. These initial priors were subsequently updated after 2 pilot runs of *n* = 145 and *n* = 294, respectively, to further increase the efficiency of the DCE design.

### Data Collection and Survey Design

The DCE was embedded in a web-based self-complete survey, and data collection took place from December 11, 2020, to March 6, 2021. The survey started with a brief description of its purpose, after which a consent form was included. After providing informed consent, participants completed the MHQoL to familiarize themselves with the attributes and levels included in the DCE tasks. Subsequently, participants were asked to complete 5 warm-up tasks that gradually introduced them to the layout and structure of the choice tasks. To minimize the potential impact of the (order of) attributes included in the warm-up tasks on the choice decisions in the actual choice tasks, 3 versions of warm-up tasks were created and randomly assigned to participants. After completing the warm-up questions, participants were assigned the actual set of 15 matched pairwise choice tasks in 3 blocks of 5 tasks. To minimize potential attribute-order effects,^23,24^ the order in which MHQoL attributes were presented was randomized, although, per individual participant, 1 constant order of attributes was used in all 15 choice tasks. In addition, randomization was also applied to the order of choice tasks within each of the 3 blocks of 5 choice tasks. After the first 2 blocks of 5 choice tasks, some standard background questions (gender, age, country of birth, marital status, education, and work status) were included to reduce DCE response fatigue. At the end of the survey, participants were asked to complete a question on life satisfaction (the Cantril Ladder of Life), the EuroQol-visual analogue scale (EQ-VAS), a question on whether COVID-19 influenced the importance placed on the MHQoL dimensions, and several survey satisfaction and cognitive debriefing questions, and they were given the opportunity to leave any comments (if any) about the survey. Data from participants were excluded if they did not complete the survey. The survey was formatted for completion on a computer or laptop. Hence, completion of the survey via a handheld device like a smartphone was not allowed. The initial survey was pilot tested through a think-aloud protocol^25^ with 3 persons who were randomly recruited by Dynata. Based on the pilot data, minor changes were made to the layout of the survey and to the instructions accompanying the warm-up questions, resulting in the final survey that was used to collect data in the main study. The full and final survey in Dutch can be found in Appendix A.

### Statistical Analyses

Demographic characteristics and feasibility outcomes (i.e., dropout rate, completion time, survey satisfaction, and cognitive debriefing questions) were examined using descriptive statistics. The completion time was calculated as the cumulative time spent on each of the pages of the survey. The utility decrements and QALY estimates^i^ for the MHQoL were obtained and conform to the conceptual framework of time-preference corrected QALY value sets, outlined in detail by Jonker et al.^16^ The model specification that was fitted was taken from the article by Jonker et al.^16^ and assumed that the utility (U_ijt_) that respondent i obtains from alternative j in choice task t is the sum of a deterministic (V_ijt_) and stochastic component (ε_ijt_):

(1) $$U_{\mathrm{ijt}}=V_{\mathrm{ijt}}+\varepsilon_{\mathrm{ijt},}$$

with the deterministic component (V_ijt_) defined as the product of the health state characteristics (X_ijt_) and accompanying preference parameters (β_i_) multiplied by the net present value (NPV_ijt_) of the number of life years (T_ijt_) spend in each health state:

(2) $$U_{\mathrm{ijt}}={\left(\beta_{i.}X_{\mathrm{ijt}}\right)}_{.}{\mathrm{NPV}}_{\mathrm{itj}}+\varepsilon_{\mathrm{ijt}.}$$

Here, X_ijt_ comprises the intercept plus *dummy-coded* MHQoL health state characteristics, and NPV_ijt_ was defined as

(3) $$\mathrm{NP}V_{\mathrm{ijt}}=\sum PV_{\left(r,s\right)},\;s=1\;\mathrm{to}\;T_{\mathrm{itj}}.$$

One of the benefits of this model specification is that any discounting function can be used to calculate the present value (PV) of future life-years; indeed, equation (3) states only that the NPV is the sum of the PVs of all future years lived and thus depends on the type of discount function, the discount rate r, and on the number of life years T.

Similar to Jonket et al. (model specification 2), nonlinear time preferences were accommodated for using the standard exponential discounting function, which is defined as:

(4) $${\mathrm{PV}}_{\left(r,s\right)}=\mathrm{EXP}\left(-{r\;}^{*}s\right).$$

After working out the summation, equation (3) combined with an exponential PV simplifies into a standard annuity with exponential discount rate r:

(5) $$\begin{array}{c} \mathrm{NP}V_{\mathrm{itj}}=T_{\mathrm{itj}\;}\;\mathrm{if}\;r=0\\ =\left(1-\exp\left(-rT_{\mathrm{itj}}\right)\right)/\left(\exp\left(r\right)-1\right)\;\mathrm{if}\;r\neq 0. \end{array}$$

Accordingly, linear time preferences were included as a special case, which occurs when the discount rate is equal to zero and thus was neither imposed nor ignored from the outset.

A random-parameter (i.e., mixed) logit model was used to analyze the choice data. This model specification assumed that the stochastic error term (ε_ijt_) was independent and identically Gumbel distributed and that the respondent-specific β-parameters were multivariate normal distributed with population mean µ and covariance matrix Σ:

(6) $$\beta_{i}\sim \mathrm{multivariate}\;\mathrm{Normal}\left(\mu ,\Sigma \right).$$

The model specification was fitted with OpenBUGS using Markov Chain Monte Carlo (MCMC) techniques. All estimations used 50,000 iterations to let 3 MCMC chains converge and 150,000 iterations to reliably approximate the posterior distributions of the health state decrements on the (latent) utility and on the 0–1 QALY scale. Decrements on the QALY scale were obtained by dividing all mean MIXL parameters by the first mean MIXL parameter:

(7) $$\mathrm{QAL}Y_{\mathrm{tariff}}=\mu /\mu_{\left(1\right)}.$$

In addition, the final value set was calculated by constraining positive decrements to 0. The statistical fit of the model was evaluated based on the McFadden *R*^2^ statistic.^26^ Successful convergence of the MCMC sampler was evaluated based on a visual inspection of the MCMC chains and the convergence diagnostics as implemented in the OpenBUGS package. The OpenBUGS model code, including the specification of the priors and computation of the QALY decrements, is included in Appendix B.

To minimize the effect of respondents with low data quality (i.e., speeders and individuals who clicked randomly through the survey), respondents with an individual-level posterior probability of the McFadden *R*^2^ being smaller or equal to zero (with posterior probability >0.10)^27^ and respondents with a DCE completion time <1.5 min, which translated into <3 s per DCE task, were removed from the final analysis.

## Results

### Characteristics of the Study Sample

Of the total of 1,828 individuals who agreed to participate in the survey and provided informed consent, 1,505 (82.3%) completed the full survey, resulting in a dropout rate of 17.7% (*n* = 323). Based on speeding and based on the posterior probabilities of the individual-level McFadden *R*^2^ being smaller than or equal to zero, 6% (*n* = 85) and 7% (*n* = 112) of respondents were removed from the final analysis, respectively, resulting in a final sample of 1,308 respondents. The average completion time of the total survey was 24 min (median = 15 min). The characteristics of the final sample are presented in Table 2. The sample is was broadly representative of the Dutch population in terms of the distribution of age and sex as recorded by Statistics Netherlands (Centraal Bureau voor de Statistiek 2020). The mean age of the sample was 47.6 y (*s* = 16.4 y), 687 (52.5%) were female, and 659 (41.6%) attained higher education. In general, participants reported high levels of quality of life as measured by the MHQoL (see Appendix C). Most participants (86%) indicated that they found the DCE choice tasks (very) clear. An overview of the responses to all cognitive debriefing questions can be found in Appendix D.

**Table 2:** Study Sample Characteristics

Characteristic	Study Sample	Sampling Quotas (Census Data)^a^
	(*N* = 1,308)	
Sex, *n* (%)		
Male	621 (47.5)	
Female	687 (52.5)	
Age, y		
Mean (*s*)	47.6 (16.4)	
Range	18–89	
Age (y), *n* (%)		
18–24	134 (10.2)	
25–34	193 (14.8)	
35–44	246 (18.8)	
45–54	249 (19.0)	
55–64	242 (18.5)	
65 and older	244 (18.7)	
Age and gender distribution, *n* (%)		
18–24, male	62 (5)	6
25–34, male	82 (6)	8
35–44, male	113 (9)	10
45–54, male	125 (10)	9
55–64, male	117 (9)	8
65 and older, male	122 (9)	9
18–24, female	72 (5)	5
25–34, female	111 (8)	8
35–44, female	133 (10)	9
45–54, female	124 (9)	9
55–64, female	125 (10)	8
65 and older	122 (9)	11
Country of birth, *n* (%)		
The Netherlands	1211 (92.6)	
Other	97 (7.4)	
Marital status, *n* (%)		
Married	606 (46.3)	
Unmarried in relationship	263 (20.1)	
Single	325 (24.8)	
Divorced	69 (5.4)	
Widowed	45 (3.4)	
Education, *n* (%)^b^		
Lower education	238 (18.3)	
Middle education	525 (40.1)	
Higher education	659 (41.6)	
Work status, *n* (%)		
Employed/self-employed	694 (53.1)	
Unemployed (able to work)	206 (15.8)	
Outside the working force (e.g., retired, student)	408 (31.1)	
Cantril Ladder of Life		
Mean (*s*)	7.2 (1.6)	
Range	0–10	
EQ-VAS		
Mean (*s*)	72.1 (21.5)	
Range	0–100	

EQ-VAS, EuroQol visual analog scale; *s*, standard deviation.

a From Statistics Netherlands (Centraal Bureau voor de Statistiek) 2020.

b Lower, middle, and higher education refers to ISCED^28^ 2011 levels 0–2 (early childhood education, primary education, lower secondary education), 3–4 (upper secondary education, post–secondary nontertiary education), and 5–8 (short-cycle tertiary education, bachelor or equivalent, master or equivalent, doctoral or equivalent), respectively.

### MHQoL Estimates

The MHQoL estimates on the latent utility and QALY scale (without and with imposed constraints) are presented in Table 3. In general, all obtained coefficients were in the expected direction and reflected the monotonic structure of the MHQoL attribute levels. Only for level 2 of the dimension “future,” the constrained model produced a slightly different coefficient than the unconstrained model did. All coefficients were statistically significant, except for the coefficients of SI2 and FU2, as their 95% credible intervals contained zero. The “physical health,”“mood,” and “relationships” dimensions were considered most important by participants. The discount rate and the McFadden *R*^2^ were, respectively, 0.148 (0.135–0.162) and 0.535 (0.525–0.547). Given the monotonic nature, the QALY scale with constraints (model 3) is the final (i.e., reference) value set. Total utility scores for the MHQoL states can be calculated by deducting the derived level estimates of the MHQoL attributes from 1. For example, the utility score for state 1122334 is 1.000 − 0.000 − 0.000 − 0.063 − 0.015 − 0.140 − 0.243 − 0.170 = 0.369. The utility values range from −0.741 for the worst state (4444444) to 1 for the best state (1111111). Based on the final value set, 19.4% of all possible states have an index value worse than death (i.e., below zero). The full value set in R, STATA, and SPSS syntax codes can be accessed via https://www.imta.nl/questionnaires/mhqol/.

**Table 3:** MHQoL Estimates (*N* = 1,308)

							Final Value Set		
	1: Latent Utility Scale			2: QALY Scale without Constraints			3: QALY Scale with Constraint		
	µ	95% CI		µ/µ_(1)_	95% CI		µ/µ_(1)_	95% CI	
		Lower	Upper		Lower	Upper		Lower	Upper
Full health	1.953	1.783	2.127	1.000	NA		1.000	NA	
SI2	−0.014	−0.037	0.009	−0.007	−0.019	0.005	−0.007	−0.019	0.005
SI3	−0.268	−0.297	−0.239	−0.137	−0.154	−0.122	−0.137	−0.154	−0.122
SI4	−0.411	−0.448	−0.375	−0.211	−0.232	−0.190	−0.211	−0.232	−0.190
IN2	−0.035	−0.058	−0.011	−0.018	−0.030	−0.006	−0.018	−0.030	−0.006
IN3	−0.230	−0.260	−0.202	−0.118	−0.134	−0.103	−0.118	−0.134	−0.103
IN4	−0.359	−0.393	−0.327	−0.184	−0.204	−0.166	−0.184	−0.204	−0.166
MO2	−0.122	−0.146	−0.099	−0.063	−0.076	−0.050	−0.063	−0.076	−0.050
MO3	−0.350	−0.384	−0.317	−0.179	−0.199	−0.161	−0.179	−0.199	−0.161
MO4	−0.606	−0.653	−0.560	−0.311	−0.340	−0.284	−0.311	−0.340	−0.284
RE2	−0.029	−0.052	−0.006	−0.015	−0.027	−0.003	−0.015	−0.027	−0.003
RE3	−0.336	−0.369	−0.303	−0.172	−0.192	−0.154	−0.172	−0.192	−0.154
RE4	−0.525	−0.568	−0.483	−0.269	−0.295	−0.245	−0.269	−0.295	−0.245
DA2	−0.042	−0.066	−0.017	−0.021	−0.034	−0.009	−0.021	−0.034	−0.009
DA3	−0.274	−0.302	−0.246	−0.140	−0.157	−0.125	−0.140	−0.157	−0.125
DA4	−0.416	−0.452	−0.381	−0.213	−0.235	−0.194	−0.213	−0.235	−0.194
PH2	−0.124	−0.149	−0.100	−0.064	−0.077	−0.051	−0.064	−0.077	−0.051
PH3	−0.474	−0.515	−0.435	−0.243	−0.267	−0.222	−0.243	−0.267	−0.222
PH4	−0.746	−0.806	−0.689	−0.383	−0.417	−0.351	−0.383	−0.417	−0.351
FU2	0.021	−0.004	0.047	0.011	−0.002	0.024	0.000	NA	
FU3	−0.206	−0.234	−0.179	−0.106	−0.121	−0.091	−0.106	−0.121	−0.091
FU4	−0.332	−0.365	−0.300	−0.170	−0.190	−0.152	−0.170	−0.190	−0.152
No. of coefficients of which their 95% credible intervals contained zero	2	NA		2	NA		1	NA	

CI, credible interval; DA, daily activities; FU, future; IN, independence; MHQoL, Mental Health Quality of Life questionnaire; MO, mood; NA, not applicable; PH, physical health; RE, relationships; SI, self-image.

## Discussion

The aim of this study was to derive a Dutch value set for the MHQoL, a quality of life instrument specifically designed for use in people with mental health problems. The applied DCE with duration design that accommodates nonlinear time preferences^17^ enabled the generation of a preference-based value set that allows for the generation of an index value on a QALY scale anchored at 0 (death) and 1 (full mental health). The utility values range from −0.741 for the worst state (4444444) to 1 for the best state (1111111).

The obtained coefficients were all in the expected direction and reflected the monotonic structure of the MHQoL, except for level 2 of the dimension “future,” for which the coefficient was (small yet) positive rather than the expected negative sign. Given that there was no logical explanation for this unexpected finding and given the fact that for use in health technology assessments and economic evaluations, a logically consistent model seems more appropriate, the coefficient of level 2 of the dimension “future” was constrained to be equal to level 1 of the dimension “future.” To assess what may have caused the found positive coefficient of level 2 of the dimension “future,” it is recommended to carry out a think-aloud study in future research. In line with related research,^29^ the most important dimensions of utility were “physical health,”“mood,” and “relationships.” The obtained discount rate of 14.8% was higher than the discount rate observed in similar studies based on different instruments,^16^ which may be related to the nature of MHQoL states or the timing of data collection. Moreover, the resulting value set was corrected for time preferences, and therefore, the exact size of the empirical discount rate does not matter for the correct interpretation and use of the value set in cost-utility analyses of mental health care interventions.

The results were robust to the exclusion of participants with seemingly inconsistent choice behavior and “speeders” (see Appendix E for the MHQoL estimates in the full sample [*N* = 1,505] and in the sample in which only the speeders were excluded [*n* = 1,420]). The results of the think-aloud study and the cognitive debriefing questions indicated that, despite the relatively large descriptive system of the MHQoL, the DCE tasks and survey as a whole were considered feasible, interesting, and could be completed within a reasonable time frame (average completion time was 24 min).

With the availability of a value set, the MHQoL may be used to generate mental health state utility values for QALY calculations in Dutch cost-utility analyses of mental health care interventions. It should, however, be noted that the choice of instruments to be used in economic evaluations to inform decisions on the collective reimbursement of health care interventions is debated. The debate in this area relates both to the sensitivity of available preference-based quality of life instruments but also to the scope of the evaluative space and comparability. To ensure the comparability of economic evaluations in health care, national health technology assessment agencies such as the Dutch National Health Care Institute prescribe the use of a generic preference-based quality of life instrument (e.g., the EQ-5D) when performing economic evaluations in health care.^30^ However, in patient populations in which the prescribed generic instrument does not capture the relevant quality of life domains or has been found to be insufficiently sensitive to condition-specific effects of interventions, alternative preference-based quality of life instruments may be used in addition to the recommended generic instrument. As the scope of the MHQoL was set in such a way to capture the most important dimensions in the context of mental health problems and services, the MHQoL is likely to be more sensitive to the benefits of mental health care interventions than the preference-based generic quality of life measures.^10^ Future research is required to compare the psychometric properties, including the sensitivity to change, of the MHQoL to other (generic) quality of life measures in the evaluation of mental health care interventions. In addition, it should be emphasized that next to the advantages of using domain-specific instruments such as the MHQoL, a disadvantage is the reduced comparability to other studies using generic (or other domain- or disease-specific) quality of life measures. It seems advisable, also to better understand their relative performance, to first use the MHQoL in combination with a generic quality of life instrument, such as the EQ-5D.

Despite the fact that the present study adopted a state-of-the-art DCE design, several limitations to this study need to be acknowledged. First, although the final sample of individuals showed a good representation of the general Dutch population in terms of the distribution of age and sex in The Netherlands, there was a slight underrepresentation of males aged 18 to 44 y and females aged 65 y and older, as respondents in these groups had an above-average risk of being identified as speeder or having used a random choice pattern. In addition, in line with previous online survey research involving voluntary participation,^31^ there was an overrepresentation of highly educated individuals. Second, although the pilot tests and cognitive debriefing questions indicated that the DCE tasks were considered feasible, the relatively large number of attributes in the DCE tasks may have been cognitively challenging and hence could have led to the adoption of simplifying choice heuristics by some participants. To minimize this potential risk, several measures, including the use of matched pairwise choice tasks and the adoption of level overlap and color coding, were taken to reduce the complexity of the choice tasks. These complexity-reducing measures, and more in specifically the combination of the use of level overlap and color coding, have previously been found to reduce the use of simplifying choice heuristics by participants.^18,19^ Third, data collection was performed during the COVID-19 pandemic, which may have altered the relative preferences of participants for the various MHQoL dimensions. Analysis of the responses to the question on whether COVID-19 influenced the importance placed on the MHQoL dimensions suggested that the dimensions “relationships” and “physical health” have become more important to more than one-third (*n* > 500) of the participants since the COVID-19 pandemic (see Appendix F). This finding raises the question if and to what extent these indicated changes affected the elicited preferences of participants and whether these changes will be permanent. These are interesting and important questions but fall beyond the scope of the current study and require attention in future research. Finally, cross-national differences in preferences for health and, hence, in the ranking of dimensions, due to, among others, possible cultural, social, and demographic differences, limit the transferability of the developed Dutch value set across countries. Hence, future studies are required to generate country-specific value sets to increase the accuracy of country-specific health economic analyses.

## Conclusions

This study derived a Dutch value set for the MHQoL by using a DCE with duration design that accommodated nonlinear time preferences. This value set allows for the generation of an index value on a QALY scale and may hence be used in Dutch cost-utility analyses of mental health care interventions. Future research is required to assess the psychometric performance of the generated value set in various patient populations and for the generation of country-specific value sets.

## Supplemental Material

sj-pdf-1-mdm-10.1177_0272989X231208645 – Supplemental material for Estimating a Preference-Based Value Set for the Mental Health Quality of Life Questionnaire (MHQoL) Click here for additional data file. Supplemental material, sj-pdf-1-mdm-10.1177_0272989X231208645 for Estimating a Preference-Based Value Set for the Mental Health Quality of Life Questionnaire (MHQoL) by Frédérique C. W. van Krugten, Marcel F. Jonker, Sebastian F. W. Himmler, Leona Hakkaart-van Roijen and Werner B. F. Brouwer in Medical Decision Making

sj-pdf-2-mdm-10.1177_0272989X231208645 – Supplemental material for Estimating a Preference-Based Value Set for the Mental Health Quality of Life Questionnaire (MHQoL) Click here for additional data file. Supplemental material, sj-pdf-2-mdm-10.1177_0272989X231208645 for Estimating a Preference-Based Value Set for the Mental Health Quality of Life Questionnaire (MHQoL) by Frédérique C. W. van Krugten, Marcel F. Jonker, Sebastian F. W. Himmler, Leona Hakkaart-van Roijen and Werner B. F. Brouwer in Medical Decision Making

sj-pdf-3-mdm-10.1177_0272989X231208645 – Supplemental material for Estimating a Preference-Based Value Set for the Mental Health Quality of Life Questionnaire (MHQoL) Click here for additional data file. Supplemental material, sj-pdf-3-mdm-10.1177_0272989X231208645 for Estimating a Preference-Based Value Set for the Mental Health Quality of Life Questionnaire (MHQoL) by Frédérique C. W. van Krugten, Marcel F. Jonker, Sebastian F. W. Himmler, Leona Hakkaart-van Roijen and Werner B. F. Brouwer in Medical Decision Making

sj-pdf-4-mdm-10.1177_0272989X231208645 – Supplemental material for Estimating a Preference-Based Value Set for the Mental Health Quality of Life Questionnaire (MHQoL) Click here for additional data file. Supplemental material, sj-pdf-4-mdm-10.1177_0272989X231208645 for Estimating a Preference-Based Value Set for the Mental Health Quality of Life Questionnaire (MHQoL) by Frédérique C. W. van Krugten, Marcel F. Jonker, Sebastian F. W. Himmler, Leona Hakkaart-van Roijen and Werner B. F. Brouwer in Medical Decision Making

sj-pdf-5-mdm-10.1177_0272989X231208645 – Supplemental material for Estimating a Preference-Based Value Set for the Mental Health Quality of Life Questionnaire (MHQoL) Click here for additional data file. Supplemental material, sj-pdf-5-mdm-10.1177_0272989X231208645 for Estimating a Preference-Based Value Set for the Mental Health Quality of Life Questionnaire (MHQoL) by Frédérique C. W. van Krugten, Marcel F. Jonker, Sebastian F. W. Himmler, Leona Hakkaart-van Roijen and Werner B. F. Brouwer in Medical Decision Making

sj-pdf-6-mdm-10.1177_0272989X231208645 – Supplemental material for Estimating a Preference-Based Value Set for the Mental Health Quality of Life Questionnaire (MHQoL) Click here for additional data file. Supplemental material, sj-pdf-6-mdm-10.1177_0272989X231208645 for Estimating a Preference-Based Value Set for the Mental Health Quality of Life Questionnaire (MHQoL) by Frédérique C. W. van Krugten, Marcel F. Jonker, Sebastian F. W. Himmler, Leona Hakkaart-van Roijen and Werner B. F. Brouwer in Medical Decision Making

## Notes

i. Note that we use the term *QALY scale* here, although the current scale arguably is conceptually different from a traditional QALY scale given that it reflects mental health–related quality of life.
