# Supplementary material for: Estimating a Preference-Based Value Set for the Mental Health Quality of Life Questionnaire (MHQoL)
Source: Med Decis Making. 2023 Nov 19;44(1):64–75. doi: 10.1177/0272989X231208645 (PMC10714713; doi:10.1177/0272989X231208645)
Supplement: sj-pdf-1-mdm-10.1177_0272989X231208645 – Supplemental material for Estimating a Preference-Based Value Set for the Mental Health Quality of Life Questionnaire (MHQoL) [file sj-pdf-1-mdm-10.1177_0272989X231208645.pdf]

Start

De Erasmus Universiteit Rotterdam doet onderzoek naar kwaliteit van leven. Met dit onderzoek willen wij inzicht krijgen in hoe belangrijk mensen verschillende aspecten vinden voor hun kwaliteit van leven. In deze vragenlijst zijn er geen goede of foute antwoorden - wij zijn benieuwd naar uw mening over wat belangrijk is voor uw kwaliteit van leven.

De vragenlijst bestaat uit drie delen en neemt tussen de 15 en 20 minuten in beslag. De resultaten zullen worden gebruikt voor wetenschappelijk onderzoek naar kwaliteit van leven. Uw antwoorden blijven anoniem. Uw deelname is vrijwillig en u kunt uw deelname aan deze vragenlijst op elk gewenst moment stoppen. Als u besluit te stoppen voor het einde van de vragenlijst, wordt alle door u verstrekte informatie verwijderd.

De gegevens die wij met de vragenlijst verzamelen worden 10 jaar bewaard en kunnen in de toekomst ook worden gebruikt voor ander onderzoek door de Erasmus Universiteit Rotterdam.

Hartelijk dank voor uw deelname!

Erasmus School of Health Policy & Management

ERASMUS UNIVERSITEIT ROTTERDAM

consent

Bevestigt u alstublieft elk van de onderstaande zaken door elk vakje aan te vinken:

consent_1 ☐ Ik begrijp dat het doel van dit onderzoek is om beter inzicht te krijgen in het relatieve belang van verschillende onderdelen van kwaliteit van leven, zoals lichamelijke gezondheid en sociale contacten.

consent_2 ☐ Ik neem vrijwillig deel aan dit onderzoek.

consent_3 ☐ Ik geef toestemming voor het verwerken van mijn (bijzondere) persoonsgegevens voor wetenschappelijk onderzoek.

consent_4 ☐ Ik geef toestemming voor het bewaren en het hergebruiken van de bij mij verzamelde onderzoeksgegevens in toekomstig onderzoek door de Erasmus Universiteit Rotterdam, in toekomstig wetenschappelijk onderzoek in andere onderzoeksvelden en voor onderwijsdoeleinden.

AGE

Wat is uw leeftijd in jaren?

infoMHQoL

Allereerst zouden we graag meer weten over uw kwaliteit van leven op dit moment.

Selecteer bij ieder onderdeel de uitspraak die uw situatie VANDAAG het best beschrijft.

MHQoL1

ZELFBEELD

MHQoL1=1 Ik denk **heel positief** over mijzelf

MHQoL1=2 Ik denk **positief** over mijzelf

MHQoL1=3 Ik denk **negatief** over mijzelf

MHQoL1=4 Ik denk **heel negatief** over mijzelf

MHQoL2

ONAFHANKELIJKHEID

Bijvoorbeeld: keuzevrijheid, financieel, meebeslissen

MHQoL2=1 Ik ben **heel tevreden** over de mate van mijn onafhankelijkheid

MHQoL2=2 Ik ben **tevreden** over de mate van mijn onafhankelijkheid

MHQoL2=3 Ik ben **ontevreden** over de mate van mijn onafhankelijkheid

MHQoL2=4 Ik ben **heel ontevreden** over de mate van mijn onafhankelijkheid

MHQoL3

STEMMING

MHQoL3=1 Ik voel mij **niet** angstig, somber of depressief

MHQoL3=2 Ik voel mij **een beetje** angstig, somber of depressief

MHQoL3=3 Ik voel mij angstig, somber of depressief

MHQoL3=4 Ik voel mij **heel** angstig, somber of depressief

infoMHQoL1

Selecteer bij ieder onderdeel de uitspraak die uw situatie VANDAAG het best beschrijft.

MHQoL4

RELATIES

Bijvoorbeeld: partner, kinderen, familie, vrienden

MHQoL4=1 Ik ben **heel tevreden** over mijn relaties

MHQoL4=2 Ik ben **tevreden** over mijn relaties

MHQoL4=3 Ik ben **ontevreden** over mijn relaties

MHQoL4=4 Ik ben **heel ontevreden** over mijn relaties

MHQoL5

DAGELIJKSE ACTIVITEITEN

Bijvoorbeeld: werk, studie, huishouden, vrijetijdsactiviteiten

MHQoL5=1 Ik ben **heel tevreden** over mijn dagelijkse activiteiten

MHQoL5=2 Ik ben **tevreden** over mijn dagelijkse activiteiten

MHQoL5=3 Ik ben **ontevreden** over mijn dagelijkse activiteiten

MHQoL5=4 Ik ben **heel ontevreden** over mijn dagelijkse activiteiten

MHQoL6

LICHAMELIJKE GEZONDHEID

MHQoL6=1 Ik heb **geen problemen** met mijn lichamelijke gezondheid

MHQoL6=2 Ik heb **enkele problemen** met mijn lichamelijke gezondheid

MHQoL6=3 Ik heb **veel problemen** met mijn lichamelijke gezondheid

MHQoL6=4 Ik heb **zeer veel problemen** met mijn lichamelijke gezondheid

MHQoL7

TOEKOMST

MHQoL7=1 Ik zie mijn toekomst **heel positief** in

MHQoL7=2 Ik zie mijn toekomst **positief** in

MHQoL7=3 Ik zie mijn toekomst **somber** in

MHQoL7=4 Ik zie mijn toekomst **heel somber** in

infowidescreen

Als u gebruik maakt van een tablet vragen we u uw tablet 90 graden te draaien naar de landschapsmodus.

Dit maakt het makkelijker om het volgende gedeelte van de vragenlijst te beantwoorden.

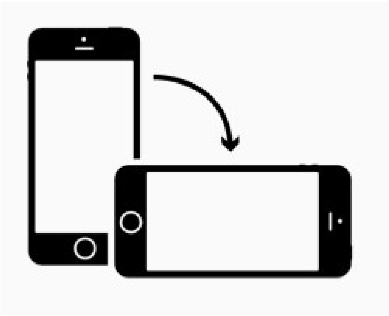

infofruit

Hierna vragen wij u enkele keuzes te maken. Het gaat bij deze vragen steeds om uw voorkeur. We beginnen met een makkelijk voorbeeld om aan de vragen te wennen.

Welk fruit vindt u lekkerder?

Klik op de knop onder de keuze die uw voorkeur heeft.

fruitDCEquestion

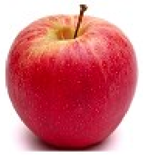

fruitDCEquestion_r1=1

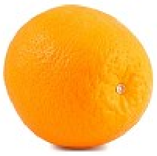

fruitDCEquestion_r1=2

Klik op de knop onder de optie die uw voorkeur heeft.

infoDCE1

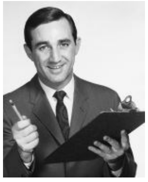

Hierna vragen wij u steeds om te kiezen tussen twee situaties die verschillen in kwaliteit van leven.

Deze situaties heeft u zelf mogelijk niet eerder meegemaakt. We vragen u daarom om u zo goed mogelijk voor te stellen hoe u de beschreven situaties zelf zou ervaren.

We vragen u om aan te geven welke situatie uw voorkeur heeft.

We beginnen met een paar voorbeelden, zodat u aan de vragen kunt wennen.

warmupFixed1

Stelt u zich het volgende voor:

In optie A zou u de rest van uw leven heel tevreden zijn over de mate van uw onafhankelijkheid en tevreden zijn over uw relaties, maar zeer veel problemen hebben met uw lichamelijke gezondheid.

In optie B zou u de rest van uw leven tevreden zijn over de mate van uw onafhankelijkheid en tevreden zijn over uw relaties, maar veel problemen hebben met uw lichamelijke gezondheid.

Welke optie heeft uw voorkeur: A of B?

	A	B
ⓘ **Onafhankelijkheid**	**Heel tevreden**	**Tevreden**
ⓘ **Relaties**	**Tevreden**	**Tevreden**
**Lichamelijke gezondheid**	**Zeer veel** problemen	**Veel** problemen
	warmupFixed1_response=1	warmupFixed1_response=2
	warmupFixed1_response	warmupFixed1_response

• Plaats uw cursor op de ⓘ voor een beschrijving van de onderdelen.

warmupFixed2

Om makkelijker het verschil te kunnen zien tussen de situaties, hebben de beschrijvingen nu verschillende kleuren:

Aspecten van kwaliteit van leven met een betere score zijn lichtpaars gekleurd en aspecten met een slechtere score donkerpaars.

Als aspecten dezelfde score hebben in optie A en B hebben ze dezelfde kleur (in dit voorbeeld tevreden zijn over uw relaties).

Welke optie heeft uw voorkeur: A of B?

	A	B
ⓘ **Onafhankelijkheid**	**Heel tevreden**	**Tevreden**
ⓘ **Relaties**	**Tevreden**	**Tevreden**
**Lichamelijke gezondheid**	**Zeer veel** problemen	**Veel** problemen
	warmupFixed2_response=1	warmupFixed2_response=2
	warmupFixed2_response	warmupFixed2_response

- Plaats uw cursor op de ⓘ voor een beschrijving van de onderdelen.

warmupFixed3

Hiervoor werd u gevraagd te kiezen tussen situaties waarin u de rest van uw leven zou doorbrengen. Vanaf nu wordt u gevraagd om te kiezen tussen situaties waarbij u nog maar een beperkt aantal jaren te leven hebt.

Bijvoorbeeld: Stelt u zich voor dat u nog 10 jaar te leven hebt en daarna zou overlijden. Welke kwaliteit van leven zou u tijdens die 10 jaar liever hebben, die in optie A of die in optie B?

• In optie A (links) zou u de komende 10 jaar heel tevreden zijn over de mate van uw onafhankelijkheid en tevreden zijn over uw relaties, maar zeer veel problemen hebben met uw lichamelijke gezondheid.

• In optie B (midden) zou u de komende 10 jaar tevreden zijn over de mate van uw onafhankelijkheid en tevreden zijn over uw relaties, maar veel problemen hebben met uw lichamelijke gezondheid.

Welke optie heeft uw voorkeur: A of B?

	A	B	
	**10 jaar** in deze situatie, gevolgd door de dood	**10 jaar** in deze situatie, gevolgd door de dood	**8 jaar** in deze situatie, gevolgd door de dood
ⓘ **Onafhankelijkheid**	**Heel tevreden**	**Tevreden**	**Heel tevreden**
ⓘ **Relaties**	**Tevreden**	**Tevreden**	**Heel tevreden**
**Lichamelijke gezondheid**	**Zeer veel** problemen	**Veel** problemen	**Geen** problemen
	warmupFixed3_response=1	warmupFixed3_response=2	
	warmupFixed3_response	warmupFixed3_response	

• Positieve onderdelen zijn **lichtpaars** en negatieve onderdelen zijn **donkerpaars**.
• Plaats uw cursor op de ⓘ voor een beschrijving van de onderdelen.

warmupFixed4

Bij iedere keuze stellen we ook een vervolgvraag. Na het kiezen tussen optie A of B, krijgt u een extra optie C. Wij vragen u dan om te kiezen tussen optie B (midden) en C (rechts). In optie B leeft u langer maar met een slechtere kwaliteit van leven. In optie C leeft u korter maar met een betere kwaliteit van leven.

Welke optie heeft uw voorkeur: B of C?

	A	B	C
	**10 jaar** in deze situatie, gevolgd door de dood	**10 jaar** in deze situatie, gevolgd door de dood	**8 jaar** in deze situatie, gevolgd door de dood
ⓘ **Onafhankelijkheid**	**Heel tevreden**	**Tevreden**	**Heel tevreden**
ⓘ **Relaties**	**Tevreden**	**Tevreden**	**Heel tevreden**
**Lichamelijke gezondheid**	**Zeer veel** problemen	**Veel** problemen	**Geen** problemen
		warmupFixed4_response=2	warmupFixed4_response=3
		warmupFixed4_response	warmupFixed4_response

- Positieve onderdelen zijn **lichtpaars** en negatieve onderdelen zijn **donkerpaars**.
- Plaats uw cursor op de ⓘ voor een beschrijving van de onderdelen.

warmup2Fixed1

Stelt u zich het volgende voor:

In optie A zou u de rest van uw leven heel positief over uzelf denken en tevreden zijn over uw dagelijkse activiteiten, maar uw toekomst heel somber inzien.

In optie B zou u de rest van uw leven positief over uzelf denken en tevreden zijn over uw dagelijkse activiteiten, maar uw toekomst somber inzien.

Welke optie heeft uw voorkeur: A of B?

	A	B
**Zelfbeeld**	**Heel positief** zelfbeeld	**Positief** zelfbeeld
ⓘ **Dagelijkse activiteiten**	**Tevreden**	**Tevreden**
**Toekomst**	Zie ik **heel somber** in	Zie ik **somber** in
	warmup2Fixed1_response=1	warmup2Fixed1_response=2
	warmup2Fixed1_response	warmup2Fixed1_response

• Plaats uw cursor op de ⓘ voor een beschrijving van de onderdelen.

warmup2Fixed2

Om makkelijker het verschil te kunnen zien tussen de situaties, hebben de beschrijvingen nu verschillende kleuren:

Aspecten van kwaliteit van leven met een betere score zijn lichtpaars gekleurd en aspecten met een slechtere score donkerpaars.

Als aspecten dezelfde score hebben in optie A en B hebben ze dezelfde kleur (in dit voorbeeld tevreden zijn over uw dagelijkse activiteiten).

Welke optie heeft uw voorkeur: A of B?

	A	B
**Zelfbeeld**	**Heel positief** zelfbeeld	**Positief** zelfbeeld
ⓘ **Dagelijkse activiteiten**	**Tevreden**	**Tevreden**
**Toekomst**	Zie ik **heel somber** in	Zie ik **somber** in
	warmup2Fixed2_response=1	warmup2Fixed2_response=2
	warmup2Fixed2_response	warmup2Fixed2_response

• Plaats uw cursor op de ⓘ voor een beschrijving van de onderdelen.

warmup2Fixed3

Hiervoor werd u gevraagd te kiezen tussen situaties waarin u de rest van uw leven zou doorbrengen. Vanaf nu wordt u gevraagd om te kiezen tussen situaties waarbij u nog maar een beperkt aantal jaren te leven hebt.

Bijvoorbeeld: Stelt u zich voor dat u nog 10 jaar te leven hebt en daarna zou overlijden. Welke kwaliteit van leven zou u tijdens die 10 jaar liever hebben, die in optie A of die in optie B?

• In optie A (links) zou u de komende 10 jaar heel positief over uzelf denken en tevreden zijn over uw dagelijkse activiteiten, maar uw toekomst heel somber inzien.

• In optie B (midden) zou u de komende 10 jaar positief over uzelf denken en tevreden zijn over uw dagelijkse activiteiten, maar uw toekomst somber inzien.

Welke optie heeft uw voorkeur: A of B?

	A	B	
	**10 jaar** in deze situatie, gevolgd door de dood	**10 jaar** in deze situatie, gevolgd door de dood	**8 jaar** in deze situatie, gevolgd door de dood
**Zelfbeeld**	**Heel positief** zelfbeeld	**Positief** zelfbeeld	**Heel positief** zelfbeeld
ⓘ **Dagelijkse activiteiten**	**Tevreden**	**Tevreden**	**Heel tevreden**
**Toekomst**	Zie ik **heel somber** in	Zie ik **somber** in	Zie ik **heel positief** in
	warmup2Fixed3_response=1	warmup2Fixed3_response=2	
	warmup2Fixed3_response	warmup2Fixed3_response	

• Positieve onderdelen zijn **lichtpaars** en negatieve onderdelen zijn **donkerpaars**.
• Plaats uw cursor op de ⓘ voor een beschrijving van de onderdelen.

warmup2Fixed4

Bij iedere keuze stellen we ook een vervolgvraag. Na het kiezen tussen optie A of B, krijgt u een extra optie C. Wij vragen u dan om te kiezen tussen optie B (midden) en C (rechts). In optie B leeft u langer maar met een slechtere kwaliteit van leven. In optie C leeft u korter maar met een betere kwaliteit van leven.

Welke optie heeft uw voorkeur: B of C?

		B	C
	**10 jaar** in deze situatie, gevolgd door de dood	**10 jaar** in deze situatie, gevolgd door de dood	**8 jaar** in deze situatie, gevolgd door de dood
**Zelfbeeld**	**Heel positief** zelfbeeld	**Positief** zelfbeeld	**Heel positief** zelfbeeld
ⓘ **Dagelijkse activiteiten**	**Tevreden**	**Tevreden**	**Heel tevreden**
**Toekomst**	Zie ik **heel somber** in	Zie ik **somber** in	Zie ik **heel positief** in
		warmup2Fixed4_response=2	warmup2Fixed4_response=3
		warmup2Fixed4_response	warmup2Fixed4_response

- Positieve onderdelen zijn **lichtpaars** en negatieve onderdelen zijn **donkerpaars**.
- Plaats uw cursor op de ⓘ voor een beschrijving van de onderdelen.

warmup3Fixed1

Stelt u zich het volgende voor:

In optie A zou u de rest van uw leven heel positief over uzelf denken, zich een beetje angstig, somber of depressief voelen en heel ontevreden zijn over uw dagelijkse activiteiten.

In optie B zou u de rest van uw leven postief over uzelf denken, zich een beetje angstig, somber of depressief voelen en ontevreden zijn over uw dagelijkse activiteiten.

Welke optie heeft uw voorkeur: A of B?

	A	B
**Zelfbeeld**	**Heel positief** zelfbeeld	**Positief** zelfbeeld
**Stemming**	**Een beetje** angstig, somber of depressief	**Een beetje** angstig, somber of depressief
ⓘ **Dagelijkse activiteiten**	**Heel ontevreden**	**Ontevreden**
	warmup3Fixed1_response=1	warmup3Fixed1_response=2
	warmup3Fixed1_response	warmup3Fixed1_response

• Plaats uw cursor op de ⓘ voor een beschrijving van de onderdelen.

warmup3Fixed2

Om makkelijker het verschil te kunnen zien tussen de situaties, hebben de beschrijvingen nu verschillende kleuren:

Aspecten van kwaliteit van leven met een betere score zijn lichtpaars gekleurd en aspecten met een slechtere score donkerpaars.

Als aspecten dezelfde score hebben in optie A en B hebben ze dezelfde kleur (in dit voorbeeld een beetje angstig, somber of depressief voelen).

Welke optie heeft uw voorkeur: A of B?

	A	B
**Zelfbeeld**	**Heel positief** zelfbeeld	**Positief** zelfbeeld
**Stemming**	**Een beetje** angstig, somber of depressief	**Een beetje** angstig, somber of depressief
ⓘ **Dagelijkse activiteiten**	**Heel ontevreden**	**Ontevreden**
	warmup3Fixed2_response=1	warmup3Fixed2_response=2
	warmup3Fixed2_response	warmup3Fixed2_response

- Plaats uw cursor op de ⓘ voor een beschrijving van de onderdelen.

warmup3Fixed3

Hiervoor werd u gevraagd te kiezen tussen situaties waarin u de rest van uw leven zou doorbrengen. Vanaf nu wordt u gevraagd om te kiezen tussen situaties waarbij u nog maar een beperkt aantal jaren te leven hebt.

Bijvoorbeeld: Stelt u zich voor dat u nog 10 jaar te leven hebt en daarna zou overlijden. Welke kwaliteit van leven zou u tijdens die 10 jaar liever hebben, die in optie A of die in optie B?

• In optie A (links) zou u de komende 10 jaar heel positief over uzelf denken, zich een beetje angstig, somber of depressief voelen en heel ontevreden zijn over uw dagelijkse activiteiten.

• In optie B (midden) zou u de komende 10 jaar positief over uzelf denken, zich een beetje angstig, somber of depressief voelen en ontevreden zijn over uw dagelijkse activiteiten.

Welke optie heeft uw voorkeur: A of B?

	A	B	
	**10 jaar** in deze situatie, gevolgd door de dood	**10 jaar** in deze situatie, gevolgd door de dood	**8 jaar** in deze situatie, gevolgd door de dood
**Zelfbeeld**	**Heel positief** zelfbeeld	**Positief** zelfbeeld	**Heel positief** zelfbeeld
**Stemming**	**Een beetje** angstig, somber of depressief	**Een beetje** angstig, somber of depressief	**Niet** angstig, somber of depressief
ⓘ **Dagelijkse activiteiten**	**Heel ontevreden**	**Ontevreden**	**Heel tevreden**
	warmup3Fixed3_response=1	warmup3Fixed3_response=2	
	warmup3Fixed3_response	warmup3Fixed3_response	

- Positieve onderdelen zijn **lichtpaars** en negatieve onderdelen zijn **donkerpaars**.
- Plaats uw cursor op de ⓘ voor een beschrijving van de onderdelen.

warmup3Fixed4

Bij iedere keuze stellen we ook een vervolgvraag. Na het kiezen tussen optie A of B, krijgt u een extra optie C. Wij vragen u dan om te kiezen tussen optie B (midden) en C (rechts). In optie B leeft u langer maar met een slechtere kwaliteit van leven. In optie C leeft u korter maar met een betere kwaliteit van leven.

Welke optie heeft uw voorkeur: B of C?

		B	C
	**10 jaar** in deze situatie, gevolgd door de dood	**10 jaar** in deze situatie, gevolgd door de dood	**8 jaar** in deze situatie, gevolgd door de dood
**Zelfbeeld**	**Heel positief** zelfbeeld	**Positief** zelfbeeld	**Heel positief** zelfbeeld
**Stemming**	**Een beetje** angstig, somber of depressief	**Een beetje** angstig, somber of depressief	**Niet** angstig, somber of depressief
ⓘ **Dagelijkse activiteiten**	**Heel ontevreden**	**Ontevreden**	**Heel tevreden**
		warmup3Fixed4_response=2	warmup3Fixed4_response=3
		warmup3Fixed4_response	warmup3Fixed4_response

- Positieve onderdelen zijn **lichtpaars** en negatieve onderdelen zijn **donkerpaars**.
- Plaats uw cursor op de ⓘ voor een beschrijving van de onderdelen.

FFFixed1

Als laatste stap in de voorbeelden beschrijven we kwaliteit van leven niet met maar drie aspecten, maar, net als in het begin, met zeven aspecten.

Bij de volgende keuzes verschillen de opties A en B steeds maar op drie van de zeven aspecten. De andere vier aspecten zijn hetzelfde in beide opties en hebben daarom dezelfde kleur.

Welke optie heeft uw voorkeur: A of B?

	A	B	
	**12 jaar** in deze situatie, gevolgd door de dood	**12 jaar** in deze situatie, gevolgd door de dood	**10 jaar** in deze situatie, gevolgd door de dood
ⓘ **Onafhankelijkheid**	**Heel tevreden**	**Heel tevreden**	**Heel tevreden**
**Lichamelijke gezondheid**	**Enkele** problemen	**Zeer veel** problemen	**Geen** problemen
**Toekomst**	Zie ik **heel positief** in	Zie ik **heel positief** in	Zie ik **heel positief** in
**Stemming**	Angstig, somber of depressief	Angstig, somber of depressief	**Niet** angstig, somber of depressief
ⓘ **Relaties**	**Heel ontevreden**	**Heel ontevreden**	**Heel tevreden**
**Zelfbeeld**	**Positief** zelfbeeld	**Heel positief** zelfbeeld	**Heel positief** zelfbeeld
ⓘ **Dagelijkse activiteiten**	**Heel ontevreden**	**Ontevreden**	**Heel tevreden**
	FFFixed1_response=1	FFFixed1_response=2	
	FFFixed1_response	FFFixed1_response	

- Positieve onderdelen zijn **lichtpaars** en negatieve onderdelen zijn **donkerpaars**.
- Plaats uw cursor op de ⓘ voor een beschrijving van de onderdelen.

infostartDCE

Nu volgen drie blokken van vijf keuzetaken, steeds met enkele algemene vragen ertussen in.

Net als in de vorige keuzetaken bestaat iedere keuzetaak uit twee delen: deel 1, waarin de keuze is tussen optie A of B (voor optie C kan dan niet worden gekozen) en deel 2, waarin de keuze is tussen optie B of C (voor optie A kan dan niet worden gekozen). Nadat u een keuze hebt gemaakt in deel 1, kunt u gelijk een keuze maken in deel 2 zonder op ''volgende'' te klikken.

Elke vraag is net iets anders; hieruit kunnen we afleiden wat u belangrijk vindt. Neem de tijd om de opties goed te bekijken en geef steeds aan welke optie u het beste vindt.

FFRandom1

[Script]

Welke optie heeft uw voorkeur: A of B? (1/5)

	A	B	
	**20 jaar** in deze situatie, gevolgd door de dood	**20 jaar** in deze situatie, gevolgd door de dood	**19 jaar** in deze situatie, gevolgd door de dood
ⓘ **Onafhankelijkheid**	**Tevreden**	**Heel tevreden**	**Heel tevreden**
**Lichamelijke gezondheid**	**Enkele** problemen	**Enkele** problemen	**Geen** problemen
**Toekomst**	Zie ik **somber** in	Zie ik **somber** in	Zie ik **heel positief** in
**Stemming**	**Niet** angstig, somber of depressief	**Niet** angstig, somber of depressief	**Niet** angstig, somber of depressief
ⓘ **Relaties**	**Heel tevreden**	**Heel tevreden**	**Heel tevreden**
**Zelfbeeld**	**Positief** zelfbeeld	**Negatief** zelfbeeld	**Heel positief** zelfbeeld
ⓘ **Dagelijkse activiteiten**	**Ontevreden**	**Tevreden**	**Heel tevreden**
	FFRandom1_response=1	FFRandom1_response=2	
	FFRandom1_response	FFRandom1_response	

- Positieve onderdelen zijn **lichtpaars** en negatieve onderdelen zijn **donkerpaars**.
- Plaats uw cursor op de ⓘ voor een beschrijving van de onderdelen.

FFbRandom1

Welke optie heeft uw voorkeur: B of C? Vergeet niet dat u korter leeft in optie C. **(1/5)**

		B	C
	**20 jaar** in deze situatie, gevolgd door de dood	**20 jaar** in deze situatie, gevolgd door de dood	**19 jaar** in deze situatie, gevolgd door de dood
ⓘ **Onafhankelijkheid**	**Tevreden**	**Heel tevreden**	**Heel tevreden**
**Lichamelijke gezondheid**	**Enkele** problemen	**Enkele** problemen	**Geen** problemen
**Toekomst**	Zie ik **somber** in	Zie ik **somber** in	Zie ik **heel positief** in
**Stemming**	**Niet** angstig, somber of depressief	**Niet** angstig, somber of depressief	**Niet** angstig, somber of depressief
ⓘ **Relaties**	**Heel tevreden**	**Heel tevreden**	**Heel tevreden**
**Zelfbeeld**	**Positief** zelfbeeld	**Negatief** zelfbeeld	**Heel positief** zelfbeeld
ⓘ **Dagelijkse activiteiten**	**Ontevreden**	**Tevreden**	**Heel tevreden**
		FFbRandom1_response=2	FFbRandom1_response=3
		FFbRandom1_response	FFbRandom1_response

- Positieve onderdelen zijn **lichtpaars** en negatieve onderdelen zijn **donkerpaars**.
- Plaats uw cursor op de ⓘ voor een beschrijving van de onderdelen.

FFRandom2

Welke optie heeft uw voorkeur: A of B? **(2/5)**

	A	B	
	**12 jaar** in deze situatie, gevolgd door de dood	**12 jaar** in deze situatie, gevolgd door de dood	**8 jaar** in deze situatie, gevolgd door de dood
	**Tevreden**	**Ontevreden**	
	**Enkele** problemen	**Enkele** problemen	
ⓘ **Onafhankelijkheid**			**Heel tevreden**
**Lichamelijke gezondheid**			**Geen** problemen
**Toekomst**	Zie ik **heel positief** in	Zie ik **heel positief** in	Zie ik **heel positief** in
**Stemming**	**Niet** angstig, somber of depressief	**Niet** angstig, somber of depressief	**Niet** angstig, somber of depressief
ⓘ **Relaties**	**Heel tevreden**	**Tevreden**	**Heel tevreden**
**Zelfbeeld**	**Positief** zelfbeeld	**Positief** zelfbeeld	**Heel positief** zelfbeeld
ⓘ **Dagelijkse activiteiten**	**Heel ontevreden**	**Tevreden**	**Heel tevreden**
	FFRandom2_response=1	FFRandom2_response=2	
	FFRandom2_response	FFRandom2_response	

- Positieve onderdelen zijn **lichtpaars** en negatieve onderdelen zijn **donkerpaars**.
- Plaats uw cursor op de ⓘ voor een beschrijving van de onderdelen.

FFbRandom2

Welke optie heeft uw voorkeur: B of C? Vergeet niet dat u korter leeft in optie C. **(2/5)**

	(grey)	B	C
	**12 jaar** in deze situatie, gevolgd door de dood	**12 jaar** in deze situatie, gevolgd door de dood	**8 jaar** in deze situatie, gevolgd door de dood
ⓘ **Onafhankelijkheid**	**Tevreden**	**Ontevreden**	**Heel tevreden**
**Lichamelijke gezondheid**	**Enkele** problemen	**Enkele** problemen	**Geen** problemen
**Toekomst**	Zie ik **heel positief** in	Zie ik **heel positief** in	Zie ik **heel positief** in
**Stemming**	**Niet** angstig, somber of depressief	**Niet** angstig, somber of depressief	**Niet** angstig, somber of depressief
ⓘ **Relaties**	**Heel tevreden**	**Tevreden**	**Heel tevreden**
**Zelfbeeld**	**Positief** zelfbeeld	**Positief** zelfbeeld	**Heel positief** zelfbeeld
ⓘ **Dagelijkse activiteiten**	**Heel ontevreden**	**Tevreden**	**Heel tevreden**
		FFbRandom2_response=2	FFbRandom2_response=3
		FFbRandom2_response	FFbRandom2_response

- Positieve onderdelen zijn **lichtpaars** en negatieve onderdelen zijn **donkerpaars**.
- Plaats uw cursor op de ⓘ voor een beschrijving van de onderdelen.

FFRandom3

Welke optie heeft uw voorkeur: A of B? **(3/5)**

	A	B	
	**8 jaar** in deze situatie, gevolgd door de dood	**8 jaar** in deze situatie, gevolgd door de dood	**2 jaar** in deze situatie, gevolgd door de dood
ⓘ **Onafhankelijkheid**	**Ontevreden**	**Ontevreden**	**Heel tevreden**
**Lichamelijke gezondheid**	**Zeer veel** problemen	**Zeer veel** problemen	**Geen** problemen
**Toekomst**	Zie ik **somber** in	Zie ik **heel somber** in	Zie ik **heel positief** in
**Stemming**	**Heel** angstig, somber of depressief	**Heel** angstig, somber of depressief	**Niet** angstig, somber of depressief
ⓘ **Relaties**	**Ontevreden**	**Heel tevreden**	**Heel tevreden**
**Zelfbeeld**	**Heel positief** zelfbeeld	**Negatief** zelfbeeld	**Heel positief** zelfbeeld
ⓘ **Dagelijkse activiteiten**	**Heel tevreden**	**Heel tevreden**	**Heel tevreden**
	FFRandom3_response=1	FFRandom3_response=2	
	FFRandom3_response	FFRandom3_response	

- Positieve onderdelen zijn **lichtpaars** en negatieve onderdelen zijn **donkerpaars**.
- Plaats uw cursor op de ⓘ voor een beschrijving van de onderdelen.

FFbRandom3

Welke optie heeft uw voorkeur: B of C? (3/5)

		B	C
	**8 jaar** in deze situatie, gevolgd door de dood	**8 jaar** in deze situatie, gevolgd door de dood	**2 jaar** in deze situatie, gevolgd door de dood
ⓘ **Onafhankelijkheid**	**Ontevreden**	**Ontevreden**	**Heel tevreden**
**Lichamelijke gezondheid**	**Zeer veel** problemen	**Zeer veel** problemen	**Geen** problemen
**Toekomst**	Zie ik **somber** in	Zie ik **heel somber** in	Zie ik **heel positief** in
**Stemming**	**Heel** angstig, somber of depressief	**Heel** angstig, somber of depressief	**Niet** angstig, somber of depressief
ⓘ **Relaties**	**Ontevreden**	**Heel tevreden**	**Heel tevreden**
**Zelfbeeld**	**Heel positief** zelfbeeld	**Negatief** zelfbeeld	**Heel positief** zelfbeeld
ⓘ **Dagelijkse activiteiten**	**Heel tevreden**	**Heel tevreden**	**Heel tevreden**
		FFbRandom3_response=2	FFbRandom3_response=3
		FFbRandom3_response	FFbRandom3_response

- Positieve onderdelen zijn **lichtpaars** en negatieve onderdelen zijn **donkerpaars**.
- Plaats uw cursor op de ⓘ voor een beschrijving van de onderdelen.

FFRandom4

Welke optie heeft uw voorkeur: A of B? **(4/5)**

	A	B	
	**14 jaar** in deze situatie, gevolgd door de dood	**14 jaar** in deze situatie, gevolgd door de dood	**7 jaar** in deze situatie, gevolgd door de dood
ⓘ **Onafhankelijkheid**	**Heel tevreden**	**Heel tevreden**	**Heel tevreden**
**Lichamelijke gezondheid**	**Geen** problemen	**Geen** problemen	**Geen** problemen
**Toekomst**	Zie ik **heel positief** in	Zie ik **somber** in	Zie ik **heel positief** in
**Stemming**	**Een beetje** angstig, somber of depressief	**Een beetje** angstig, somber of depressief	**Niet** angstig, somber of depressief
ⓘ **Relaties**	**Heel ontevreden**	**Heel tevreden**	**Heel tevreden**
**Zelfbeeld**	**Heel positief** zelfbeeld	**Heel negatief** zelfbeeld	**Heel positief** zelfbeeld
ⓘ **Dagelijkse activiteiten**	**Ontevreden**	**Ontevreden**	**Heel tevreden**
	FFRandom4_response=1	FFRandom4_response=2	
	FFRandom4_response	FFRandom4_response	

- Positieve onderdelen zijn **lichtpaars** en negatieve onderdelen zijn **donkerpaars**.
- Plaats uw cursor op de ⓘ voor een beschrijving van de onderdelen.

FFbRandom4

Welke optie heeft uw voorkeur: B of C? **(4/5)**

		B	C
	**14 jaar** in deze situatie, gevolgd door de dood	**14 jaar** in deze situatie, gevolgd door de dood	**7 jaar** in deze situatie, gevolgd door de dood
ⓘ **Onafhankelijkheid**	**Heel tevreden**	**Heel tevreden**	**Heel tevreden**
**Lichamelijke gezondheid**	**Geen** problemen	**Geen** problemen	**Geen** problemen
**Toekomst**	Zie ik **heel positief** in	Zie ik **somber** in	Zie ik **heel positief** in
**Stemming**	**Een beetje** angstig, somber of depressief	**Een beetje** angstig, somber of depressief	**Niet** angstig, somber of depressief
ⓘ **Relaties**	**Heel ontevreden**	**Heel tevreden**	**Heel tevreden**
**Zelfbeeld**	**Heel positief** zelfbeeld	**Heel negatief** zelfbeeld	**Heel positief** zelfbeeld
ⓘ **Dagelijkse activiteiten**	**Ontevreden**	**Ontevreden**	**Heel tevreden**
		FFbRandom4_response=2	FFbRandom4_response=3
		FFbRandom4_response	FFbRandom4_response

- Positieve onderdelen zijn **lichtpaars** en negatieve onderdelen zijn **donkerpaars**.
- Plaats uw cursor op de ⓘ voor een beschrijving van de onderdelen.

FFRandom5

Welke optie heeft uw voorkeur: A of B? **(5/5)**

	A	B	
	**2 jaar** in deze situatie, gevolgd door de dood	**2 jaar** in deze situatie, gevolgd door de dood	**0,25 jaar** in deze situatie, gevolgd door de dood
	**Heel ontevreden**	**Ontevreden**	
	**Veel** problemen	**Zeer veel** problemen	
ⓘ **Onafhankelijkheid**	Zie ik **positief** in	Zie ik **positief** in	**Heel tevreden**
**Lichamelijke gezondheid**	**Een beetje** angstig, somber of depressief	**Een beetje** angstig, somber of depressief	**Geen** problemen
**Toekomst**	**Heel ontevreden**	**Heel ontevreden**	Zie ik **heel positief** in
**Stemming**	**Heel positief** zelfbeeld	**Positief** zelfbeeld	**Niet** angstig, somber of depressief
ⓘ **Relaties**	**Heel ontevreden**	**Heel ontevreden**	**Heel tevreden**
**Zelfbeeld**	FFRandom5_response=1	FFRandom5_response=2	**Heel positief** zelfbeeld
ⓘ **Dagelijkse activiteiten**			**Heel tevreden**
	FFRandom5_response	FFRandom5_response	

- Positieve onderdelen zijn **lichtpaars** en negatieve onderdelen zijn **donkerpaars**.
- Plaats uw cursor op de ⓘ voor een beschrijving van de onderdelen.

FFbRandom5

Welke optie heeft uw voorkeur: B of C? (5/5)

		B	C
	**2 jaar** in deze situatie, gevolgd door de dood	**2 jaar** in deze situatie, gevolgd door de dood	**0,25 jaar** in deze situatie, gevolgd door de dood
ⓘ **Onafhankelijkheid**	**Heel ontevreden**	**Ontevreden**	**Heel tevreden**
**Lichamelijke gezondheid**	**Veel** problemen	**Zeer veel** problemen	**Geen** problemen
**Toekomst**	Zie ik **positief** in	Zie ik **positief** in	Zie ik **heel positief** in
**Stemming**	**Een beetje** angstig, somber of depressief	**Een beetje** angstig, somber of depressief	**Niet** angstig, somber of depressief
ⓘ **Relaties**	**Heel ontevreden**	**Heel ontevreden**	**Heel tevreden**
**Zelfbeeld**	**Heel positief** zelfbeeld	**Positief** zelfbeeld	**Heel positief** zelfbeeld
ⓘ **Dagelijkse activiteiten**	**Heel ontevreden**	**Heel ontevreden**	**Heel tevreden**
		FFbRandom5_response=2	FFbRandom5_response=3
		FFbRandom5_response	FFbRandom5_response

- Positieve onderdelen zijn **lichtpaars** en negatieve onderdelen zijn **donkerpaars**.
- Plaats uw cursor op de ⓘ voor een beschrijving van de onderdelen.

intermediate1

U hebt het eerste blok van vijf keuzetaken afgerond.

Voor u verder gaat met het tweede blok van vijf keuzetaken willen we u vragen enkele algemene vragen over uzelf te beantwoorden.

Gender

Wat is uw geslacht?

Gender=1 Man

Gender=2 Vrouw

Gender=3 Gender_3_other
Anders, namelijk: ____

MARITALSTATUS

Wat is uw burgerlijke staat?

MARITALSTATUS=1 Gehuwd (wettig gehuwd of geregistreerd partnerschap)

MARITALSTATUS=2 Ongehuwd in relatie, samenwonend

MARITALSTATUS=3 Ongehuwd in relatie, niet samenwonend

MARITALSTATUS=4 Alleenstaand

MARITALSTATUS=5 Gescheiden

MARITALSTATUS=6 Weduwe/weduwnaar

Nu volgt het tweede blok van vijf keuzetaken.

FFRandom6

Welke optie heeft uw voorkeur: A of B? **(1/5)**

	A	B	
	**10 jaar** in deze situatie, gevolgd door de dood	**10 jaar** in deze situatie, gevolgd door de dood	**5 jaar** in deze situatie, gevolgd door de dood
ⓘ **Onafhankelijkheid**	**Tevreden**	**Ontevreden**	**Heel tevreden**
**Lichamelijke gezondheid**	**Veel** problemen	**Veel** problemen	**Geen** problemen
**Toekomst**	Zie ik **positief** in	Zie ik **positief** in	Zie ik **heel positief** in
**Stemming**	**Niet** angstig, somber of depressief	**Niet** angstig, somber of depressief	**Niet** angstig, somber of depressief
ⓘ **Relaties**	**Ontevreden**	**Heel ontevreden**	**Heel tevreden**
**Zelfbeeld**	**Heel negatief** zelfbeeld	**Heel negatief** zelfbeeld	**Heel positief** zelfbeeld
ⓘ **Dagelijkse activiteiten**	**Tevreden**	**Heel tevreden**	**Heel tevreden**
	FFRandom6_response=1	FFRandom6_response=2	
	FFRandom6_response	FFRandom6_response	

- Positieve onderdelen zijn **lichtpaars** en negatieve onderdelen zijn **donkerpaars**.
- Plaats uw cursor op de ⓘ voor een beschrijving van de onderdelen.

FFbRandom6

Welke optie heeft uw voorkeur: B of C? Vergeet niet dat u korter leeft in optie C. **(1/5)**

	A	B	C
	**10 jaar** in deze situatie, gevolgd door de dood	**10 jaar** in deze situatie, gevolgd door de dood	**5 jaar** in deze situatie, gevolgd door de dood
ⓘ **Onafhankelijkheid**	**Tevreden**	**Ontevreden**	**Heel tevreden**
**Lichamelijke gezondheid**	**Veel** problemen	**Veel** problemen	**Geen** problemen
**Toekomst**	Zie ik **positief** in	Zie ik **positief** in	Zie ik **heel positief** in
**Stemming**	**Niet** angstig, somber of depressief	**Niet** angstig, somber of depressief	**Niet** angstig, somber of depressief
ⓘ **Relaties**	**Ontevreden**	**Heel ontevreden**	**Heel tevreden**
**Zelfbeeld**	**Heel negatief** zelfbeeld	**Heel negatief** zelfbeeld	**Heel positief** zelfbeeld
ⓘ **Dagelijkse activiteiten**	**Tevreden**	**Heel tevreden**	**Heel tevreden**
		FFbRandom6_response=2	FFbRandom6_response=3
		FFbRandom6_response	FFbRandom6_response

• Positieve onderdelen zijn **lichtpaars** en negatieve onderdelen zijn **donkerpaars**.
• Plaats uw cursor op de ⓘ voor een beschrijving van de onderdelen.

FFRandom7

Welke optie heeft uw voorkeur: A of B? **(2/5)**

	A	B	
	**4 jaar** in deze situatie, gevolgd door de dood	**4 jaar** in deze situatie, gevolgd door de dood	**0,5 jaar** in deze situatie, gevolgd door de dood
ⓘ **Onafhankelijkheid**	**Heel tevreden**	**Heel ontevreden**	**Heel tevreden**
**Lichamelijke gezondheid**	**Veel** problemen	**Veel** problemen	**Geen** problemen
**Toekomst**	Zie ik **heel somber** in	Zie ik **heel somber** in	Zie ik **heel positief** in
**Stemming**	Angstig, somber of depressief	**Een beetje** angstig, somber of depressief	**Niet** angstig, somber of depressief
ⓘ **Relaties**	**Ontevreden**	**Ontevreden**	**Heel tevreden**
**Zelfbeeld**	**Heel negatief** zelfbeeld	**Negatief** zelfbeeld	**Heel positief** zelfbeeld
ⓘ **Dagelijkse activiteiten**	**Tevreden**	**Tevreden**	**Heel tevreden**
	FFRandom7_response=1	FFRandom7_response=2	
	FFRandom7_response	FFRandom7_response	

- Positieve onderdelen zijn **lichtpaars** en negatieve onderdelen zijn **donkerpaars**.
- Plaats uw cursor op de ⓘ voor een beschrijving van de onderdelen.

FFbRandom7

Welke optie heeft uw voorkeur: B of C? **(2/5)**

		B	C
	**4 jaar** in deze situatie, gevolgd door de dood	**4 jaar** in deze situatie, gevolgd door de dood	**0,5 jaar** in deze situatie, gevolgd door de dood
ⓘ **Onafhankelijkheid**	**Heel tevreden**	**Heel ontevreden**	**Heel tevreden**
**Lichamelijke gezondheid**	**Veel** problemen	**Veel** problemen	**Geen** problemen
**Toekomst**	Zie ik **heel somber** in	Zie ik **heel somber** in	Zie ik **heel positief** in
**Stemming**	Angstig, somber of depressief	**Een beetje** angstig, somber of depressief	**Niet** angstig, somber of depressief
ⓘ **Relaties**	**Ontevreden**	**Ontevreden**	**Heel tevreden**
**Zelfbeeld**	**Heel negatief** zelfbeeld	**Negatief** zelfbeeld	**Heel positief** zelfbeeld
ⓘ **Dagelijkse activiteiten**	**Tevreden**	**Tevreden**	**Heel tevreden**
		FFbRandom7_response=2	FFbRandom7_response=3
		FFbRandom7_response	FFbRandom7_response

- Positieve onderdelen zijn **lichtpaars** en negatieve onderdelen zijn **donkerpaars**.
- Plaats uw cursor op de ⓘ voor een beschrijving van de onderdelen.

FFRandom8

Welke optie heeft uw voorkeur: A of B? **(3/5)**

	A	B	
	**5 jaar** in deze situatie, gevolgd door de dood	**5 jaar** in deze situatie, gevolgd door de dood	**4 jaar** in deze situatie, gevolgd door de dood
ⓘ **Onafhankelijkheid**	**Heel ontevreden**	**Heel ontevreden**	**Heel tevreden**
**Lichamelijke gezondheid**	**Enkele** problemen	**Zeer veel** problemen	**Geen** problemen
**Toekomst**	Zie ik **heel somber** in	Zie ik **heel somber** in	Zie ik **heel positief** in
**Stemming**	Angstig, somber of depressief	**Niet** angstig, somber of depressief	**Niet** angstig, somber of depressief
ⓘ **Relaties**	**Heel ontevreden**	**Heel ontevreden**	**Heel tevreden**
**Zelfbeeld**	**Heel positief** zelfbeeld	**Heel positief** zelfbeeld	**Heel positief** zelfbeeld
ⓘ **Dagelijkse activiteiten**	**Heel tevreden**	**Tevreden**	**Heel tevreden**
	FFRandom8_response=1	FFRandom8_response=2	
	FFRandom8_response	FFRandom8_response	

- Positieve onderdelen zijn **lichtpaars** en negatieve onderdelen zijn **donkerpaars**.
- Plaats uw cursor op de ⓘ voor een beschrijving van de onderdelen.

FFbRandom8

Welke optie heeft uw voorkeur: B of C? **(3/5)**

		B	C
	**5 jaar** in deze situatie, gevolgd door de dood	**5 jaar** in deze situatie, gevolgd door de dood	**4 jaar** in deze situatie, gevolgd door de dood
ⓘ **Onafhankelijkheid**	**Heel ontevreden**	**Heel ontevreden**	**Heel tevreden**
**Lichamelijke gezondheid**	**Enkele** problemen	**Zeer veel** problemen	**Geen** problemen
**Toekomst**	Zie ik **heel somber** in	Zie ik **heel somber** in	Zie ik **heel positief** in
**Stemming**	Angstig, somber of depressief	**Niet** angstig, somber of depressief	**Niet** angstig, somber of depressief
ⓘ **Relaties**	**Heel ontevreden**	**Heel ontevreden**	**Heel tevreden**
**Zelfbeeld**	**Heel positief** zelfbeeld	**Heel positief** zelfbeeld	**Heel positief** zelfbeeld
ⓘ **Dagelijkse activiteiten**	**Heel tevreden**	**Tevreden**	**Heel tevreden**
		FFbRandom8_response=2	FFbRandom8_response=3
		FFbRandom8_response	FFbRandom8_response

- Positieve onderdelen zijn **lichtpaars** en negatieve onderdelen zijn **donkerpaars**.
- Plaats uw cursor op de ⓘ voor een beschrijving van de onderdelen.

FFRandom9

Welke optie heeft uw voorkeur: A of B? **(4/5)**

	A	B	
	**3 jaar** in deze situatie, gevolgd door de dood	**3 jaar** in deze situatie, gevolgd door de dood	**1 jaar** in deze situatie, gevolgd door de dood
ⓘ **Onafhankelijkheid**	**Heel tevreden**	**Heel tevreden**	**Heel tevreden**
**Lichamelijke gezondheid**	**Zeer veel** problemen	**Veel** problemen	**Geen** problemen
**Toekomst**	Zie ik **heel somber** in	Zie ik **heel somber** in	Zie ik **heel positief** in
**Stemming**	**Niet** angstig, somber of depressief	Angstig, somber of depressief	**Niet** angstig, somber of depressief
ⓘ **Relaties**	**Tevreden**	**Tevreden**	**Heel tevreden**
**Zelfbeeld**	**Negatief** zelfbeeld	**Negatief** zelfbeeld	**Heel positief** zelfbeeld
ⓘ **Dagelijkse activiteiten**	**Heel ontevreden**	**Ontevreden**	**Heel tevreden**
	FFRandom9_response=1	FFRandom9_response=2	
	FFRandom9_response	FFRandom9_response	

- Positieve onderdelen zijn **lichtpaars** en negatieve onderdelen zijn **donkerpaars**.
- Plaats uw cursor op de ⓘ voor een beschrijving van de onderdelen.

FFbRandom9

Welke optie heeft uw voorkeur: B of C? **(4/5)**

	(ghosted)	B	C
	**3 jaar** in deze situatie, gevolgd door de dood	**3 jaar** in deze situatie, gevolgd door de dood	**1 jaar** in deze situatie, gevolgd door de dood
ⓘ **Onafhankelijkheid**	**Heel tevreden**	**Heel tevreden**	**Heel tevreden**
**Lichamelijke gezondheid**	**Zeer veel** problemen	**Veel** problemen	**Geen** problemen
**Toekomst**	Zie ik **heel somber** in	Zie ik **heel somber** in	Zie ik **heel positief** in
**Stemming**	**Niet** angstig, somber of depressief	Angstig, somber of depressief	**Niet** angstig, somber of depressief
ⓘ **Relaties**	**Tevreden**	**Tevreden**	**Heel tevreden**
**Zelfbeeld**	**Negatief** zelfbeeld	**Negatief** zelfbeeld	**Heel positief** zelfbeeld
ⓘ **Dagelijkse activiteiten**	**Heel ontevreden**	**Ontevreden**	**Heel tevreden**
		FFbRandom9_response=2	FFbRandom9_response=3
		FFbRandom9_response	FFbRandom9_response

- Positieve onderdelen zijn **lichtpaars** en negatieve onderdelen zijn **donkerpaars**.
- Plaats uw cursor op de ⓘ voor een beschrijving van de onderdelen.

FFRandom10

Welke optie heeft uw voorkeur: A of B? **(5/5)**

	A	B	
	**16 jaar** in deze situatie, gevolgd door de dood	**16 jaar** in deze situatie, gevolgd door de dood	**10 jaar** in deze situatie, gevolgd door de dood
ⓘ **Onafhankelijkheid**	**Heel tevreden**	**Heel tevreden**	**Heel tevreden**
**Lichamelijke gezondheid**	**Veel** problemen	**Enkele** problemen	**Geen** problemen
**Toekomst**	Zie ik **somber** in	Zie ik **heel somber** in	Zie ik **heel positief** in
**Stemming**	**Een beetje** angstig, somber of depressief	**Niet** angstig, somber of depressief	**Niet** angstig, somber of depressief
ⓘ **Relaties**	**Ontevreden**	**Ontevreden**	**Heel tevreden**
**Zelfbeeld**	**Positief** zelfbeeld	**Positief** zelfbeeld	**Heel positief** zelfbeeld
ⓘ **Dagelijkse activiteiten**	**Heel tevreden**	**Heel tevreden**	**Heel tevreden**
	FFRandom10_response=1	FFRandom10_response=2	
	FFRandom10_response	FFRandom10_response	

- Positieve onderdelen zijn **lichtpaars** en negatieve onderdelen zijn **donkerpaars**.
- Plaats uw cursor op de ⓘ voor een beschrijving van de onderdelen.

FFbRandom10

Welke optie heeft uw voorkeur: B of C? **(5/5)**

		B	C
	**16 jaar** in deze situatie, gevolgd door de dood	**16 jaar** in deze situatie, gevolgd door de dood	**10 jaar** in deze situatie, gevolgd door de dood
ⓘ **Onafhankelijkheid**	**Heel tevreden**	**Heel tevreden**	**Heel tevreden**
**Lichamelijke gezondheid**	**Veel** problemen	**Enkele** problemen	**Geen** problemen
**Toekomst**	Zie ik **somber** in	Zie ik **heel somber** in	Zie ik **heel positief** in
**Stemming**	**Een beetje** angstig, somber of depressief	**Niet** angstig, somber of depressief	**Niet** angstig, somber of depressief
ⓘ **Relaties**	**Ontevreden**	**Ontevreden**	**Heel tevreden**
**Zelfbeeld**	**Positief** zelfbeeld	**Positief** zelfbeeld	**Heel positief** zelfbeeld
ⓘ **Dagelijkse activiteiten**	**Heel tevreden**	**Heel tevreden**	**Heel tevreden**
		FFbRandom10_response=2	FFbRandom10_response=3
		FFbRandom10_response	FFbRandom10_response

- Positieve onderdelen zijn **lichtpaars** en negatieve onderdelen zijn **donkerpaars**.
- Plaats uw cursor op de ⓘ voor een beschrijving van de onderdelen.

U hebt het tweede blok van vijf keuzetaken afgerond.

Voor u verder gaat met het derde en laatste blok van vijf keuzetaken willen we u vragen enkele algemene vragen over uzelf te beantwoorden.

EDUCATION

Wat is de hoogst genoten opleiding afgesloten met certificaat of diploma?

EDUCATION=1 Geen opleiding

EDUCATION=2 LO (lagere school, basisonderwijs, LAVO, VGLO)

EDUCATION=3 LBO (VMBO basis/kader, LBO, LTS, ITO, LEAO, Huishoudschool, LLO)

EDUCATION=4 MAO (VMBO GL/TL, MAVO, IVO, MULO, ULO, 3jr HBS, 3jr VWO, 3jr VHMO)

EDUCATION=5 MBO (MTS, UTS, MEAO, ROC)

EDUCATION=6 HAO (HAVO, VWO, Atheneum, Gymnasium, NMS, HBS, Lyceum)

EDUCATION=7 HBO (HTS, HEAO, Wetensch. kand., Univers. onderwijs kand., Bachelor)

EDUCATION=8 WO (Universitair onderwijs, Doctoraalopleiding, TH, Master)

EDUCATION=9 EDUCATION_9_other

Andere (bedrijfs)opleiding of cursus, namelijk: ____

PRIMARYOCCUPATION

Welke omschrijving past het beste bij u?

PRIMARYOCCUPATION=1 Werkende met betaald werk

PRIMARYOCCUPATION=2 Werkloos of werkzoekende

PRIMARYOCCUPATION=3 Vrijwilliger

PRIMARYOCCUPATION=4 Arbeidsongeschikt

PRIMARYOCCUPATION=5 Scholier of studerende

PRIMARYOCCUPATION=6 Huisman of huisvader/huisvrouw of huismoeder

PRIMARYOCCUPATION=7 Gepensioneerd of met vervroegd pensioen

PRIMARYOCCUPATION=8 PRIMARYOCCUPATION_8_other

Anders, namelijk: ____

ETNICITY

In welk land bent u geboren?

ETNICITY=1 Nederland

ETNICITY=2 Marokko

ETNICITY=3 Turkije

ETNICITY=4 Suriname

ETNICITY=5 Voormalige Nederlandse Antillen (Curaçao, St. Maarten, Bonaire, Saba, St. Eustatius) of Aruba

ETNICITY=6 Indonesië of Molukken

ETNICITY=7 Oost-Europa

ETNICITY=8 China

ETNICITY=9 ETNICITY_9_other
Anders, namelijk: ______

Nu volgt het derde en laatste blok van vijf keuzetaken.

FFRandom11

Welke optie heeft uw voorkeur: A of B? Vergeet niet dat u korter leeft in optie C. **(1/5)**

	A	B	
	**6 jaar** in deze situatie, gevolgd door de dood	**6 jaar** in deze situatie, gevolgd door de dood	**3 jaar** in deze situatie, gevolgd door de dood
ⓘ **Onafhankelijkheid**	**Tevreden**	**Heel tevreden**	**Heel tevreden**
**Lichamelijke gezondheid**	**Enkele** problemen	**Veel** problemen	**Geen** problemen
**Toekomst**	Zie ik **heel somber** in	Zie ik **heel somber** in	Zie ik **heel positief** in
**Stemming**	**Heel** angstig, somber of depressief	**Heel** angstig, somber of depressief	**Niet** angstig, somber of depressief
ⓘ **Relaties**	**Heel ontevreden**	**Heel ontevreden**	**Heel tevreden**
**Zelfbeeld**	**Heel negatief** zelfbeeld	**Heel negatief** zelfbeeld	**Heel positief** zelfbeeld
ⓘ **Dagelijkse activiteiten**	**Tevreden**	**Heel ontevreden**	**Heel tevreden**
	FFRandom11_response=1	FFRandom11_response=2	
	FFRandom11_response	FFRandom11_response	

- Positieve onderdelen zijn **lichtpaars** en negatieve onderdelen zijn **donkerpaars**.
- Plaats uw cursor op de ⓘ voor een beschrijving van de onderdelen.

FFbRandom11

Welke optie heeft uw voorkeur: B of C? **(1/5)**

		B	C
	**6 jaar** in deze situatie, gevolgd door de dood	**6 jaar** in deze situatie, gevolgd door de dood	**3 jaar** in deze situatie, gevolgd door de dood
ⓘ **Onafhankelijkheid**	**Tevreden**	**Heel tevreden**	**Heel tevreden**
**Lichamelijke gezondheid**	**Enkele** problemen	**Veel** problemen	**Geen** problemen
**Toekomst**	Zie ik **heel somber** in	Zie ik **heel somber** in	Zie ik **heel positief** in
**Stemming**	**Heel** angstig, somber of depressief	**Heel** angstig, somber of depressief	**Niet** angstig, somber of depressief
ⓘ **Relaties**	**Heel ontevreden**	**Heel ontevreden**	**Heel tevreden**
**Zelfbeeld**	**Heel negatief** zelfbeeld	**Heel negatief** zelfbeeld	**Heel positief** zelfbeeld
ⓘ **Dagelijkse activiteiten**	**Tevreden**	**Heel ontevreden**	**Heel tevreden**
		FFbRandom11_response=2	FFbRandom11_response=3
		FFbRandom11_response	FFbRandom11_response

- Positieve onderdelen zijn **lichtpaars** en negatieve onderdelen zijn **donkerpaars**.
- Plaats uw cursor op de ⓘ voor een beschrijving van de onderdelen.

FFRandom12

Welke optie heeft uw voorkeur: A of B? **(2/5)**

	A	B	
	**10 jaar** in deze situatie, gevolgd door de dood	**10 jaar** in deze situatie, gevolgd door de dood	**6 jaar** in deze situatie, gevolgd door de dood
ⓘ **Onafhankelijkheid**	**Tevreden**	**Heel ontevreden**	**Heel tevreden**
**Lichamelijke gezondheid**	**Geen** problemen	**Geen** problemen	**Geen** problemen
**Toekomst**	Zie ik **positief** in	Zie ik **positief** in	Zie ik **heel positief** in
**Stemming**	Angstig, somber of depressief	Angstig, somber of depressief	**Niet** angstig, somber of depressief
ⓘ **Relaties**	**Tevreden**	**Heel tevreden**	**Heel tevreden**
**Zelfbeeld**	**Positief** zelfbeeld	**Positief** zelfbeeld	**Heel positief** zelfbeeld
ⓘ **Dagelijkse activiteiten**	**Heel ontevreden**	**Ontevreden**	**Heel tevreden**
	FFRandom12_response=1	FFRandom12_response=2	
	FFRandom12_response	FFRandom12_response	

- Positieve onderdelen zijn **lichtpaars** en negatieve onderdelen zijn **donkerpaars**.
- Plaats uw cursor op de ⓘ voor een beschrijving van de onderdelen.

FFbRandom12

Welke optie heeft uw voorkeur: B of C? **(2/5)**

		B	C
	**10 jaar** in deze situatie, gevolgd door de dood	**10 jaar** in deze situatie, gevolgd door de dood	**6 jaar** in deze situatie, gevolgd door de dood
ⓘ **Onafhankelijkheid**	**Tevreden**	**Heel ontevreden**	**Heel tevreden**
**Lichamelijke gezondheid**	**Geen** problemen	**Geen** problemen	**Geen** problemen
**Toekomst**	Zie ik **positief** in	Zie ik **positief** in	Zie ik **heel positief** in
**Stemming**	Angstig, somber of depressief	Angstig, somber of depressief	**Niet** angstig, somber of depressief
ⓘ **Relaties**	**Tevreden**	**Heel tevreden**	**Heel tevreden**
**Zelfbeeld**	**Positief** zelfbeeld	**Positief** zelfbeeld	**Heel positief** zelfbeeld
ⓘ **Dagelijkse activiteiten**	**Heel ontevreden**	**Ontevreden**	**Heel tevreden**
		FFbRandom12_response=2	FFbRandom12_response=3
		FFbRandom12_response	FFbRandom12_response

- Positieve onderdelen zijn **lichtpaars** en negatieve onderdelen zijn **donkerpaars**.
- Plaats uw cursor op de ⓘ voor een beschrijving van de onderdelen.

FFRandom13

Welke optie heeft uw voorkeur: A of B? **(3/5)**

	A	B	
	**18 jaar** in deze situatie, gevolgd door de dood	**18 jaar** in deze situatie, gevolgd door de dood	**11 jaar** in deze situatie, gevolgd door de dood
ⓘ **Onafhankelijkheid**	**Heel ontevreden**	**Heel tevreden**	**Heel tevreden**
**Lichamelijke gezondheid**	**Geen** problemen	**Zeer veel** problemen	**Geen** problemen
**Toekomst**	Zie ik **heel positief** in	Zie ik **heel positief** in	Zie ik **heel positief** in
**Stemming**	**Heel** angstig, somber of depressief	Angstig, somber of depressief	**Niet** angstig, somber of depressief
ⓘ **Relaties**	**Ontevreden**	**Ontevreden**	**Heel tevreden**
**Zelfbeeld**	**Negatief** zelfbeeld	**Negatief** zelfbeeld	**Heel positief** zelfbeeld
ⓘ **Dagelijkse activiteiten**	**Heel tevreden**	**Heel tevreden**	**Heel tevreden**
	FFRandom13_response=1	FFRandom13_response=2	
	FFRandom13_response	FFRandom13_response	

- Positieve onderdelen zijn **lichtpaars** en negatieve onderdelen zijn **donkerpaars**.
- Plaats uw cursor op de ⓘ voor een beschrijving van de onderdelen.

FFbRandom13

Welke optie heeft uw voorkeur: B of C? **(3/5)**

		B	C
	**18 jaar** in deze situatie, gevolgd door de dood	**18 jaar** in deze situatie, gevolgd door de dood	**11 jaar** in deze situatie, gevolgd door de dood
ⓘ **Onafhankelijkheid**	**Heel ontevreden**	**Heel tevreden**	**Heel tevreden**
**Lichamelijke gezondheid**	**Geen** problemen	**Zeer veel** problemen	**Geen** problemen
**Toekomst**	Zie ik **heel positief** in	Zie ik **heel positief** in	Zie ik **heel positief** in
**Stemming**	**Heel** angstig, somber of depressief	Angstig, somber of depressief	**Niet** angstig, somber of depressief
ⓘ **Relaties**	**Ontevreden**	**Ontevreden**	**Heel tevreden**
**Zelfbeeld**	**Negatief** zelfbeeld	**Negatief** zelfbeeld	**Heel positief** zelfbeeld
ⓘ **Dagelijkse activiteiten**	**Heel tevreden**	**Heel tevreden**	**Heel tevreden**

FFbRandom13_response=2 FFbRandom13_response=3

FFbRandom13_response FFbRandom13_response

- Positieve onderdelen zijn **lichtpaars** en negatieve onderdelen zijn **donkerpaars**.
- Plaats uw cursor op de ⓘ voor een beschrijving van de onderdelen.

FFRandom14

Welke optie heeft uw voorkeur: A of B? **(4/5)**

	A	B	
	**19 jaar** in deze situatie, gevolgd door de dood	**19 jaar** in deze situatie, gevolgd door de dood	**14 jaar** in deze situatie, gevolgd door de dood
ⓘ **Onafhankelijkheid**	**Heel tevreden**	**Heel tevreden**	**Heel tevreden**
**Lichamelijke gezondheid**	**Enkele** problemen	**Zeer veel** problemen	**Geen** problemen
**Toekomst**	Zie ik **positief** in	Zie ik **positief** in	Zie ik **heel positief** in
**Stemming**	**Heel** angstig, somber of depressief	**Heel** angstig, somber of depressief	**Niet** angstig, somber of depressief
ⓘ **Relaties**	**Heel ontevreden**	**Tevreden**	**Heel tevreden**
**Zelfbeeld**	**Positief** zelfbeeld	**Heel positief** zelfbeeld	**Heel positief** zelfbeeld
ⓘ **Dagelijkse activiteiten**	**Tevreden**	**Tevreden**	**Heel tevreden**

FFRandom14_response=1 FFRandom14_response=2

FFRandom14_response FFRandom14_response

- Positieve onderdelen zijn **lichtpaars** en negatieve onderdelen zijn **donkerpaars**.
- Plaats uw cursor op de ⓘ voor een beschrijving van de onderdelen.

FFbRandom14

Welke optie heeft uw voorkeur: B of C? (4/5)

		B	C
	**19 jaar** in deze situatie, gevolgd door de dood	**19 jaar** in deze situatie, gevolgd door de dood	**14 jaar** in deze situatie, gevolgd door de dood
ⓘ **Onafhankelijkheid**	**Heel tevreden**	**Heel tevreden**	**Heel tevreden**
**Lichamelijke gezondheid**	**Enkele** problemen	**Zeer veel** problemen	**Geen** problemen
**Toekomst**	Zie ik **positief** in	Zie ik **positief** in	Zie ik **heel positief** in
**Stemming**	**Heel** angstig, somber of depressief	**Heel** angstig, somber of depressief	**Niet** angstig, somber of depressief
ⓘ **Relaties**	**Heel ontevreden**	**Tevreden**	**Heel tevreden**
**Zelfbeeld**	**Positief** zelfbeeld	**Heel positief** zelfbeeld	**Heel positief** zelfbeeld
ⓘ **Dagelijkse activiteiten**	**Tevreden**	**Tevreden**	**Heel tevreden**
		FFbRandom14_response=2	FFbRandom14_response=3
		FFbRandom14_response	FFbRandom14_response

• Positieve onderdelen zijn **lichtpaars** en negatieve onderdelen zijn **donkerpaars**.
• Plaats uw cursor op de ⓘ voor een beschrijving van de onderdelen.

FFRandom15

Welke optie heeft uw voorkeur: A of B? **(5/5)**

	A	B	
	**17 jaar** in deze situatie, gevolgd door de dood	**17 jaar** in deze situatie, gevolgd door de dood	**15 jaar** in deze situatie, gevolgd door de dood
ⓘ **Onafhankelijkheid**	**Ontevreden**	**Heel ontevreden**	**Heel tevreden**
**Lichamelijke gezondheid**	**Enkele** problemen	**Enkele** problemen	**Geen** problemen
**Toekomst**	Zie ik **positief** in	Zie ik **positief** in	Zie ik **heel positief** in
**Stemming**	**Een beetje** angstig, somber of depressief	**Een beetje** angstig, somber of depressief	**Niet** angstig, somber of depressief
ⓘ **Relaties**	**Ontevreden**	**Ontevreden**	**Heel tevreden**
**Zelfbeeld**	**Heel positief** zelfbeeld	**Heel negatief** zelfbeeld	**Heel positief** zelfbeeld
ⓘ **Dagelijkse activiteiten**	**Heel ontevreden**	**Heel tevreden**	**Heel tevreden**
	FFRandom15_response=1	FFRandom15_response=2	
	FFRandom15_response	FFRandom15_response	

- Positieve onderdelen zijn **lichtpaars** en negatieve onderdelen zijn **donkerpaars**.
- Plaats uw cursor op de ⓘ voor een beschrijving van de onderdelen.

FFbRandom15

Welke optie heeft uw voorkeur: B of C? (5/5)

		B	C
	**17 jaar** in deze situatie, gevolgd door de dood	**17 jaar** in deze situatie, gevolgd door de dood	**15 jaar** in deze situatie, gevolgd door de dood
ⓘ **Onafhankelijkheid**	**Ontevreden**	**Heel ontevreden**	**Heel tevreden**
**Lichamelijke gezondheid**	**Enkele** problemen	**Enkele** problemen	**Geen** problemen
**Toekomst**	Zie ik **positief** in	Zie ik **positief** in	Zie ik **heel positief** in
**Stemming**	**Een beetje** angstig, somber of depressief	**Een beetje** angstig, somber of depressief	**Niet** angstig, somber of depressief
ⓘ **Relaties**	**Ontevreden**	**Ontevreden**	**Heel tevreden**
**Zelfbeeld**	**Heel positief** zelfbeeld	**Heel negatief** zelfbeeld	**Heel positief** zelfbeeld
ⓘ **Dagelijkse activiteiten**	**Heel ontevreden**	**Heel tevreden**	**Heel tevreden**
		FFbRandom15_response=2	FFbRandom15_response=3
		FFbRandom15_response	FFbRandom15_response

- Positieve onderdelen zijn **lichtpaars** en negatieve onderdelen zijn **donkerpaars**.
- Plaats uw cursor op de ⓘ voor een beschrijving van de onderdelen.

evalDCElearningtext

U hebt alle keuzetaken beantwoord.

Wat vond u van de keuzetaken?

evalDCElearning

	Helemaal mee oneens		Mee eens noch mee oneens		Helemaal mee eens
**De vragen waren duidelijk**	evalDCElearning_r1=1	evalDCElearning_r1=2	evalDCElearning_r1=3	evalDCElearning_r1=4	evalDCElearning_r1
**De verschillen tussen de beschreven situaties waren duidelijk**	evalDCElearning_r2=1	evalDCElearning_r2=2	evalDCElearning_r2=3	evalDCElearning_r2=4	evalDCElearning_r2
**Ik kon mij goed concentreren op alle vragen**	evalDCElearning_r3=1	evalDCElearning_r3=2	evalDCElearning_r3=3	evalDCElearning_r3=4	evalDCElearning_r3
**Ik heb alle onderdelen vergeleken voordat ik mijn keuze maakte**	evalDCElearning_r4=1	evalDCElearning_r4=2	evalDCElearning_r4=3	evalDCElearning_r4=4	evalDCElearning_r4

COVIDdirection

Denkt u dat de coronacrisis uw keuzes heeft beïnvloed? Vindt u bijvoorbeeld bepaalde aspecten meer of minder belangrijk geworden voor uw kwaliteit van leven dan voor de uitbraak van het coronavirus?

	Minder belangrijk	Even belangrijk	Belangrijker
Zelfbeeld	COVIDdirection_r1=1	COVIDdirection_r1=2	COVIDdirection_r1=3
ⓘ Onafhankelijkheid	COVIDdirection_r2=1	COVIDdirection_r2=2	COVIDdirection_r2=3
Stemming	COVIDdirection_r3=1	COVIDdirection_r3=2	COVIDdirection_r3=3
ⓘ Relaties	COVIDdirection_r4=1	COVIDdirection_r4=2	COVIDdirection_r4=3
ⓘ Dagelijkse activiteiten	COVIDdirection_r5=1	COVIDdirection_r5=2	COVIDdirection_r5=3
Lichamelijke gezondheid	COVIDdirection_r6=1	COVIDdirection_r6=2	COVIDdirection_r6=3
Toekomst	COVIDdirection_r7=1	COVIDdirection_r7=2	COVIDdirection_r7=3

COVID19direction

Kunt u in een paar zinnen beschrijven waarom deze aspecten meer of minder belangrijk zijn geworden voor uw kwaliteit van leven dan voor de coronacrisis?

infolastquestions

Tot slot volgen nu twee vragen over uw algemene welzijn en gezondheid.

Cantril

Uw geluk

Hier ziet u een afbeelding van een ladder. Stel dat de bovenkant van de ladder staat voor het best mogelijke leven voor u en de onderkant van de ladder het slechts denkbare leven voor u.

Geef aan waar u vindt dat u zelf op dit moment op deze ladder staat.

Best mogelijke leven

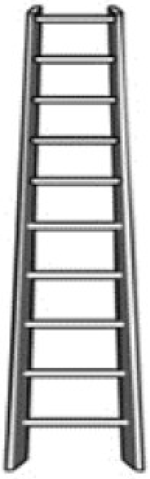

Slechtst mogelijke leven

Cantril_number=1
Cantril_number
10

Cantril_number=2
Cantril_number
9

Cantril_number=3
Cantril_number
8

Cantril_number=4
Cantril_number
7

Cantril_number=5
Cantril_number
6

Cantril_number=6
Cantril_number
5

Cantril_number=7
Cantril_number
4

Cantril_number=8
Cantril_number
3

Cantril_number=9
Cantril_number
2

Cantril_number=10
Cantril_number
1

Cantril_number=11
Cantril_number
0

EQVAS

Uw gezondheid

[illegible]

- We willen weten hoe goed of slecht uw gezondheid VANDAAG is.
- Deze meetschaal loopt van 0 tot 100.
- 100 staat voor de beste gezondheid die u zich kunt voorstellen.
- 0 staat voor de slechtste gezondheid die u zich kunt voorstellen.
- Voer het nummer in waar u uw gezondheid op de meetschaal zou plaatsen.

UW GEZONDHEID VANDAAG: EQVAS_number

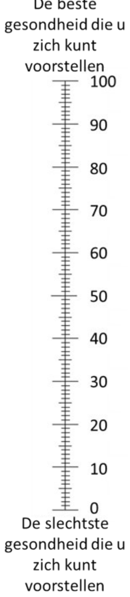

qualitativeQoL

Kunt u hieronder in een paar zinnen omschrijven wat kwaliteit van leven voor u betekent?

evalend

Wat vond u van het onderzoek in het algemeen?

	Helemaal mee oneens		Mee eens noch mee oneens		Helemaal mee eens
**Het onderwerp van de vragenlijst was interessant**	evalend_r1=1	evalend_r1=2	evalend_r1=3	evalend_r1=4	evalend_r1=5
**Het was leuk om deel te nemen aan het onderzoek**	evalend_r2=1	evalend_r2=2	evalend_r2=3	evalend_r2=4	evalend_r2=5

evalendopen

Heeft u nog opmerkingen over de keuzetaken of de vragenlijst?

endtext

Dit is het einde van de vragenlijst. Nogmaals hartelijk dank voor uw bijdrage! Klik op volgende om de vragenlijst af te ronden.

redscr

Note:

When respondents take the survey in regular mode this page will not be displayed. Respondents will be redirected to the link below:

http://dkr1.ssisurveys.com/projects/end?rst=1&basic=27863&PSID=XXXX
