# Supplementary material for: Estimating a Preference-Based Value Set for the Mental Health Quality of Life Questionnaire (MHQoL)
Source: Med Decis Making. 2023 Nov 19;44(1):64–75. doi: 10.1177/0272989X231208645 (PMC10714713; doi:10.1177/0272989X231208645)
Supplement: sj-pdf-2-mdm-10.1177_0272989X231208645 – Supplemental material for Estimating a Preference-Based Value Set for the Mental Health Quality of Life Questionnaire (MHQoL) [file sj-pdf-2-mdm-10.1177_0272989X231208645.pdf]

Appendix B – OpenBUGS model code

```
model {

# N = number of respondents
# T = number of choice tasks per respondent
# A = number of alternatives per choice task
# V = number of explanatory variables (including non-linear time preference)

# likelihood
for (n in 1:N){
 for (t in 1:T){
  Y[n,t] ~ dcat(prob[n, t, 1:2])
}}

# prob calculations <- user-written softmax function
for (n in 1:N){
 for (t in 1:T){
  prob[n,t,1:2] <- softmaxExp(X[n,t,1,], Q[n,t,1], X[n,t,2,], Q[n,t,2], beta[n,], rate)
}}


# priors

# multivariate normal prior on beta
for (n in 1:N){ beta[n,1:V] ~ dmnorm(mu_beta[], prec_beta[,]) }
mu_beta[1:V] ~ dmnorm(hyper_mu_beta[],hyper_tau_beta[,])
prec_beta[1:V,1:V] ~ dwish(scaleMatrix[,],V)

for (b in 1:V){
 hyper_mu_beta[b] <- 0
 for (bb in 1:V){
   scaleMatrix[b,bb] <- equals(b,bb)
   hyper_tau_beta[b,bb] <- equals(b,bb)/100
}}

# normal prior on discount rate
rate ~ dnorm(0,0.01)
```

```
# additional computations

# population SD
covar[1:V,1:V] <- inverse(prec_beta[,])
for (v in 1:V){ SD[v] <- sqrt(covar[v,v]) }

# log-likelihood
for (n in 1:N){
 for (t in 1:T) { LL_task[n,t] <- log( prob[n,t, Y[n,t] ]) }
 LL_resp[n] <- sum(LL_task[n,])
}
LL <- sum(LL_resp[])

# McFadden R-squared
LL_random <- N*T*log(0.5)
Rsq <- (LL - LL_random)/-LL_random

# QALY estimates
QALY[1] <- 1
for (v in 2:V){
  QALY[v] <- mu_beta[v] / mu_beta[1]
}

}
```
