# Supplementary material for: Estimating a Preference-Based Value Set for the Mental Health Quality of Life Questionnaire (MHQoL)
Source: Med Decis Making. 2023 Nov 19;44(1):64–75. doi: 10.1177/0272989X231208645 (PMC10714713; doi:10.1177/0272989X231208645)
Supplement: sj-pdf-3-mdm-10.1177_0272989X231208645 – Supplemental material for Estimating a Preference-Based Value Set for the Mental Health Quality of Life Questionnaire (MHQoL) [file sj-pdf-3-mdm-10.1177_0272989X231208645.pdf]

Appendix C – Respondents' levels of quality of life as measured by the MHQoL

Figure. Distribution of responses to the MHQoL dimensions ranging from 1 (best) to 4 (worst) (N=1,308).

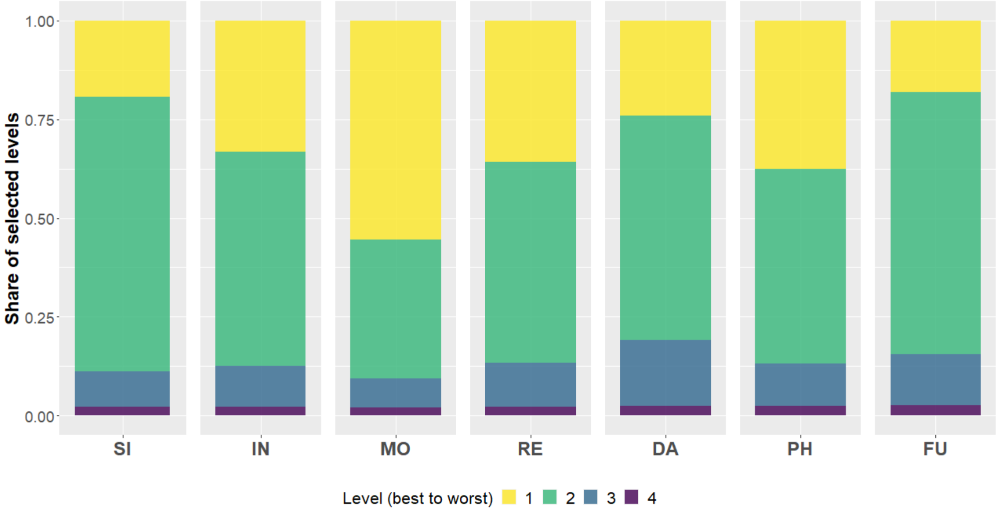

MHQoL, Mental Health Quality of Life questionnaire; SI, Self-image; IN, Independence; MO, Mood; RE, Relationships; DA, Daily activities; PH, Physical health; FU, Future.
