# Supplementary material for: Estimating a Preference-Based Value Set for the Mental Health Quality of Life Questionnaire (MHQoL)
Source: Med Decis Making. 2023 Nov 19;44(1):64–75. doi: 10.1177/0272989X231208645 (PMC10714713; doi:10.1177/0272989X231208645)
Supplement: sj-pdf-4-mdm-10.1177_0272989X231208645 – Supplemental material for Estimating a Preference-Based Value Set for the Mental Health Quality of Life Questionnaire (MHQoL) [file sj-pdf-4-mdm-10.1177_0272989X231208645.pdf]

Appendix D – Results DCE debriefing questions

Table. Responses to DCE debriefing questions (N=1.308), %.

	Completely disagree		Neither agree nor disagree		Completely agree
The subject of the survey was interesting	2.3	3.7	10.4	31.2	52.4
I enjoyed participating in the survey	1.8	4.8	13.6	35.5	44.3
The questions were clear	1.8	3.7	8.6	33.4	52.5
The differences between the described scenarios were clear	2.1	2.1	6.2	28.4	61.2
I was able to concentrate in all questions	2.3	2.6	12.6	32.7	49.8
I compared all different aspects before making my choice	1.6	3.4	14.2	33.9	46.9
