# Supplementary material for: Estimating a Preference-Based Value Set for the Mental Health Quality of Life Questionnaire (MHQoL)
Source: Med Decis Making. 2023 Nov 19;44(1):64–75. doi: 10.1177/0272989X231208645 (PMC10714713; doi:10.1177/0272989X231208645)
Supplement: sj-pdf-5-mdm-10.1177_0272989X231208645 – Supplemental material for Estimating a Preference-Based Value Set for the Mental Health Quality of Life Questionnaire (MHQoL) [file sj-pdf-5-mdm-10.1177_0272989X231208645.pdf]

Appendix E – Results additional analyses

Table 1. MHQoL estimates in full sample (N=1,505).

	**1: Latent utility-scale**			**2: QALY scale without constraints**		
	β	95% CI		β	95% CI	
		Lower	Upper		Lower	Upper
Full health	1.693	1.548	1.842	1.000	NA	
SI2	-0.003	-0.024	0.019	-0.002	-0.014	0.011
SI3	-0.228	-0.255	-0.202	-0.135	-0.152	-0.119
SI4	-0.349	-0.383	-0.316	-0.206	-0.228	-0.186
IN2	-0.022	-0.044	-4.842	-0.013	-0.026	-2.872
IN3	-0.197	-0.224	-0.171	-0.117	-0.134	-0.101
IN4	-0.306	-0.337	-0.276	-0.181	-0.201	-0.163
MO2	-0.087	-0.109	-0.065	-0.052	-0.065	-0.038
MO3	-0.295	-0.325	-0.265	-0.174	-0.194	-0.156
MO4	-0.506	-0.549	-0.465	-0.300	-0.327	-0.274
RE2	-0.010	-0.031	0.011	-0.006	-0.019	0.007
RE3	-0.280	-0.310	-0.251	-0.166	-0.185	-0.148
RE4	-0.442	-0.481	-0.404	-0.261	-0.286	-0.238
DA2	-0.022	-0.044	0.001	-0.013	-0.026	0.001
DA3	-0.227	-0.253	-0.202	-0.134	-0.151	-0.118
DA4	-0.352	-0.385	-0.320	-0.208	-0.229	-0.188
PH2	-0.097	-0.119	-0.074	-0.057	-0.071	-0.044
PH3	-0.401	-0.437	-0.366	-0.237	-0.260	-0.216
PH4	-0.625	-0.677	-0.575	-0.370	-0.402	-0.340
FU2	0.033	0.009	0.057	0.019	0.006	0.034
FU3	-0.166	-0.192	-0.140	-0.098	-0.114	-0.082
FU4	-0.274	-0.304	-0.244	-0.162	-0.182	-0.143

CI = Credible Interval; DA = Daily activities; FU = Future; IN = Independence; MHQoL = Mental Health Quality of Life questionnaire; MO = Mood; NA = Not Applicable; PH = Physical health; RE = Relationships; SI = Self-image.

Table 2. MHQoL estimates in sample in which based on speeding (i.e., DCE completion time <1.5 minute) 6% (N=85) of respondents were removed (N=1,420).

	**1: Latent utility-scale**			**2: QALY scale without constraints**		
	β	95% CI		β	95% CI	
		Lower	Upper		Lower	Upper
Full health	1.772	1.623	1.925	1.000	NA	
SI2	-0.006	-0.028	0.017	-0.003	-0.016	0.009
SI3	-0.241	-0.269	-0.215	-0.136	-0.153	-0.120
SI4	-0.372	-0.407	-0.339	-0.210	-0.232	-0.190
IN2	-0.024	-0.046	-0.002	-0.014	-0.026	-0.001
IN3	-0.210	-0.237	-0.184	-0.119	-0.135	-0.103
IN4	-0.325	-0.356	-0.295	-0.184	-0.204	-0.165
MO2	-0.102	-0.125	-0.078	-0.057	-0.071	-0.044
MO3	-0.315	-0.346	-0.285	-0.178	-0.198	-0.160
MO4	-0.544	-0.587	-0.502	-0.307	-0.335	-0.281
RE2	-0.019	-0.041	0.003	-0.011	-0.023	0.002
RE3	-0.301	-0.332	-0.272	-0.170	-0.190	-0.152
RE4	-0.473	-0.512	-0.435	-0.267	-0.292	-0.244
DA2	-0.024	-0.047	3.315	-0.013	-0.027	1.853
DA3	-0.243	-0.270	-0.216	-0.137	-0.154	-0.121
DA4	-0.373	-0.407	-0.341	-0.211	-0.232	-0.191
PH2	-0.103	-0.127	-0.080	-0.058	-0.072	-0.045
PH3	-0.425	-0.462	-0.389	-0.240	-0.263	-0.219
PH4	-0.671	-0.724	-0.620	-0.379	-0.412	-0.349
FU2	0.033	0.009	0.057	0.018	0.005	0.032
FU3	-0.179	-0.205	-0.153	-0.101	-0.117	-0.086

FU4	-0.294	-0.325	-0.263	-0.166	-0.185	-0.148

CI = Credible Interval; DA = Daily activities; FU = Future; IN = Independence; MHQoL = Mental Health Quality of Life questionnaire; MO = Mood; NA = Not Applicable; PH = Physical health; RE = Relationships; SI = Self-image.
