# Supplementary material for: Estimating a Preference-Based Value Set for the Mental Health Quality of Life Questionnaire (MHQoL)
Source: Med Decis Making. 2023 Nov 19;44(1):64–75. doi: 10.1177/0272989X231208645 (PMC10714713; doi:10.1177/0272989X231208645)

Appendix F – Direction of impact of COVID-19 pandemic on importance of MHQoL dimensions

Figure. Direction of impact of COVID-19 pandemic on importance of MHQoL dimensions (N=1,308).

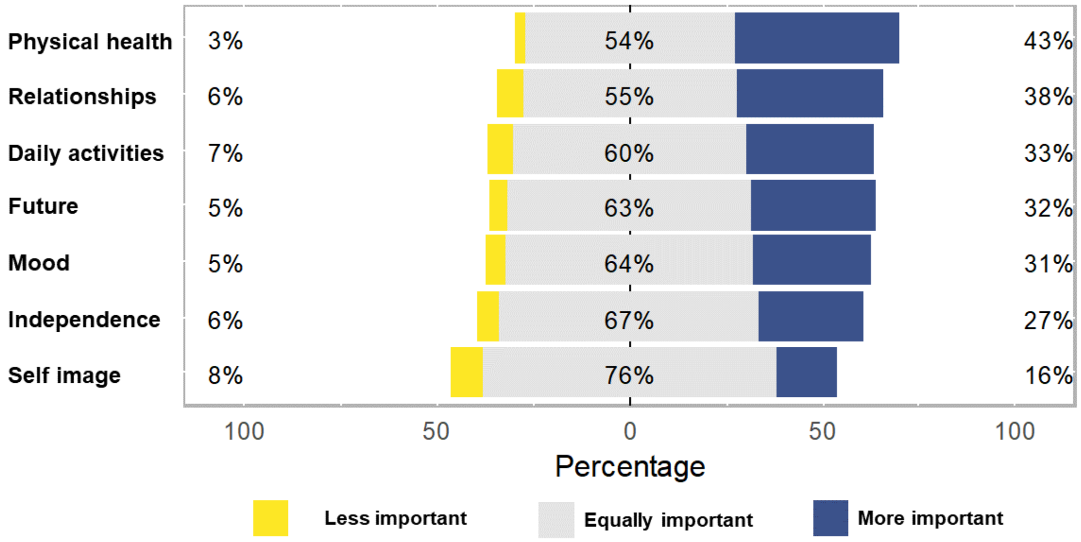

Supplement: sj-pdf-6-mdm-10.1177_0272989X231208645 – Supplemental material for Estimating a Preference-Based Value Set for the Mental Health Quality of Life Questionnaire (MHQoL) [file sj-pdf-6-mdm-10.1177_0272989X231208645.pdf]
